# Highly diverse recombining populations of *Vibrio cholerae* and *Vibrio parahaemolyticus* in French Mediterranean coastal lagoons

**DOI:** 10.3389/fmicb.2015.00708

**Published:** 2015-07-16

**Authors:** Kévin Esteves, Thomas Mosser, Fabien Aujoulat, Dominique Hervio-Heath, Patrick Monfort, Estelle Jumas-Bilak

**Affiliations:** ^1^Team “Pathogènes Hydriques Santé, Environnements”, HydroSciences Montpellier, UMR 5569, Centre National de la Recherche, IRD, Université de MontpellierMontpellier, France; ^2^Ifremer, RBE, SG2M, Laboratoire Santé, Environnement et MicrobiologiePlouzané, France; ^3^Département d'Hygiène Hospitalière, Centre Hospitalier UniversitaireMontpellier, France

**Keywords:** *Vibrio*, human pathogens, French coastal lagoons, phylogeny, recombination, multi-locus sequence analysis, virulence factor

## Abstract

*Vibrio parahaemolyticus* and *Vibrio cholerae* are ubiquitous to estuarine and marine environments. These two species found in Mediterranean coastal systems can induce infections in humans. Environmental isolates of *V. cholerae* (*n* = 109) and *V. parahaemolyticus* (*n* = 89) sampled at different dates, stations and water salinities were investigated for virulence genes and by a multilocus sequence-based analysis (MLSA). *V. cholerae* isolates were all *ctxA* negative and only one isolate of *V. parahaemolyticus* displayed *trh2* gene. Most Sequence Types (ST) corresponded to unique ST isolated at one date or one station. Frequent recombination events were detected among different pathogenic species, *V. parahaemolyticus, V. cholerae, Vibrio mimicus*, and *Vibrio metoecus*. Recombination had a major impact on the diversification of lineages. The genetic diversity assessed by the number of ST/strain was higher in low salinity condition for *V. parahaemolyticus* and *V. cholerae* whereas the frequency of recombination events in *V. cholerae* was lower in low salinity condition. Mediterranean coastal lagoon systems housed *V. cholerae* and *V. parahaemolyticus* with genetic diversities equivalent to the worldwide diversity described so far. The presence of STs found in human infections as well as the frequency of recombination events in environmental vibrios populations could predict a potential epidemiological risk.

## Introduction

The genus *Vibrio* groups gram-negative halophilic bacteria that are found in marine and estuarine environments, among which some species are responsible for human infections (Colwell et al., 1977). Particularly, *V. cholerae* pandemic serogroups O1 and O139 cause the choleric form of the disease. Many other O serogroups have been reported for cases of *V. cholerae* associated with diarrhea (Octavia et al., 2013) but the pandemic serogroups O1 and O139 are distinguished by the presence of two genetic elements, the lysogenic bacteriophage designated CTXΦ containing the genes encoding cholera toxin and the *Vibrio* Pathogenicity Island (VPI) (Sack et al., 2004). Most of *V. cholerae* isolated from the environment belong to non-toxigenic non-O1/non-O139 serotypes, which have been involved in cases of diarrhea (Dutta et al., 2013), some of them carrying various repertories of virulence genes (Octavia et al., 2013). The majority of environmental strains of *Vibrio parahaemolyticus* are innocuous but subpopulations are pathogens for humans. Potentially virulent strains are characterized by the presence of the thermostable direct (*tdh*) and/or *tdh*-related (*trh1* and *trh2*) hemolysin genes. Some pathogenic clones encountered an epidemic success that led to the dissemination of the pandemic O3:K6 clone in many countries (Nair et al., 2007) and to the worldwide emergence of other clones such as O4:K8, O4:K11, O4:K12, and O4:K13 as shown in Peru (Gavilan et al., 2013). These vibrios, especially *V. parahaemolyticus*, are recognized worldwide as agents of gastroenteritis resulting from consumption of raw or undercooked seafood (Tantillo et al., 2004; Pruzzo et al., 2005).

In most temperate coastal areas, dynamics of vibrios are seasonal. Temperature, salinity and phytoplankton, and zooplankton populations are major factors determining the abundance of vibrios (Johnson et al., 2012). The increase of *Vibrio* densities and the spread of pathogenic species in coastal marine systems have been linked to climate anomalies and in particular, to the increase of sea surface temperature (Vezzulli et al., 2013). *V. cholerae* non-O1/non-O139 and *V. parahaemolyticus* are found in coastal lagoons of Southern France (Mediterranean) (Cantet et al., 2013). Interfacing between watersheds and the sea, these transitional aquatic ecosystems are exposed to important seasonal variations of temperature and to sudden and intense Mediterranean rainfalls that lead to flash floods bringing large volumes of freshwater into lagoons, thus reducing their salinity (Pecqueur et al., 2011).

The population structure of vibrios has been increasingly studied by the means of multilocus genetics. MLST schemes and databases are available for *V. cholerae* (Teh et al., 2011; Islam et al., 2013; Octavia et al., 2013) and *V. parahaemolyticus* (Chowdhury et al., 2004a,b; Gonzalez-Escalona et al., 2008; Yan et al., 2011). Most studies on *V. cholerae* and *V. parahaemolyticus* populations are centered on human pathogenicity and/or shellfish contamination. Consequently, research efforts are concentrated on geographic regions of cholera endemicity or vibriosis outbreaks, and on pandemic serotypes or strains harboring virulence traits. It is now obvious that the ecology of potentially pathogenic vibrios extends beyond the human body (Haley et al., 2012, 2014; Islam et al., 2013) and that these vibrios do not require human transmission for persistence (Schuster et al., 2011; Haley et al., 2012). Beside investigation of vibriosis outbreaks (Chowdhury et al., 2004a; Marin et al., 2013), several MLST studies on *Vibrio* spp. considered strain collections of very diverse geographical origins and thus gave a snapshot of the global structure of *Vibrio* populations (Octavia et al., 2013) or compared clinical and environmental populations (Teh et al., 2011; Yan et al., 2011; Theethakaew et al., 2013; Turner et al., 2013; Zhang et al., 2014).

Studies involving environmental *V. cholerae* non-O1/non-O139 in cholera-free regions (Zo et al., 2009; Keymer and Boehm, 2011; Schuster et al., 2011; Octavia et al., 2013) and *V. parahaemolyticus* (Gamble and Lovell, 2011; Ellis et al., 2012; Urmersbach et al., 2014) are more recent. Findings support that some pathogenicity factors in vibrios may have an adaptive role in the natural environment (Haley et al., 2012; Islam et al., 2013). Furthermore, more and more human clinical infections are caused by non-toxigenic non-O1/non-O139 *V. cholerae* (Chatterjee et al., 2009; Hasan et al., 2012; Luo et al., 2013) and by *V. parahaemolyticus* isolates lacking *tdh, trh* genes or the type three secretion system (Chao et al., 2009; Garcia et al., 2009; Harth et al., 2009; Marin et al., 2013). From a genetic point of view, relatedness in housekeeping gene sequences between some toxigenic and non-toxigenic strains provides clues to the emergence of toxigenic strains from non-toxigenic progenitors by the acquisition of virulence genes as suggested for *V. cholerae* (Mohapatra et al., 2009; Zhou et al., 2014) and for *V. parahaemolyticus* (Chao et al., 2011; Ellingsen et al., 2013; Gavilan et al., 2013). The emergence of successful pathogenic clones from environmental populations was suspected for both species (Moore et al., 2014; Velazquez-Roman et al., 2014). All these findings suggest that, beside successful pandemic and harmful strains, environmental *V. cholerae* and *V. parahaemolyticus* species behave as non-specialist pathogens with great capacities for genetic exchange. It is therefore interesting to study populations of *V. cholerae* and *V. parahaemolyticus* sampled independently from their pathogenic behavior and/or from their virulence genes repertory.

In the present work, we focused on the genetic structure and dynamics of *V. parahaemolyticus* and *V. cholerae* populations collected in coastal lagoons in the South of France (Cantet et al., 2013) in a context of local environmental changes. The genetic diversity and the recombination rate in these two populations were considered independently from any recent history of cholera or *Vibrio* outbreaks. The effects of salinity and sampling site on genetic diversity and on intra- and interspecific recombinations were particularly studied.

## Materials and methods

### Area of study and sampling

**Figure 1:**
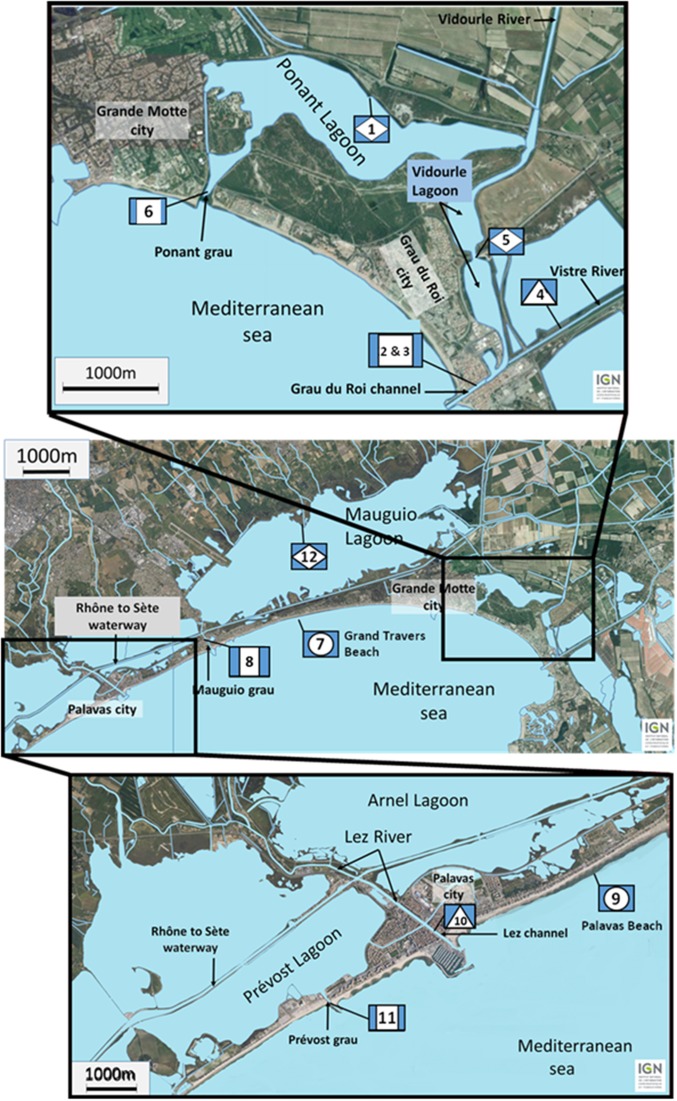
**Location of the sampling stations along the French Mediterranean coast (Languedoc area) in lagoons (diamond) (1, 5, and 12), in channels connecting lagoons (square) (2, 3, 6, 8, and 11) or rivers (triangle) (4 and 10) to the sea, and on beaches (circle) (7 and 9)**.

The studied lagoon system is presented in Figure 1. The lagoons are supplied with fresh water from four main watersheds. To the east, the water from the large watershed of the Vidourle River (826 km^2^) and its tributary the Vistre River (598 km^2^) goes into the Ponant and Vidourle lagoons before flowing to the sea via the Ponant grau and the Grau du Roi channel, respectively. Numerous streams flow directly into the Mauguio or the Prévost lagoons, forming the watersheds of Mauguio and Palavas lagoons, respectively. To the west, the Lez watershed (535 km^2^) is formed by the Lez River and by its main tributary the Mosson River. Fresh water from Lez watershed doesn't flow directly into the Prévost lagoons but cross them via the Lez channel. In the latter system, salinity variations during heavy rainfalls are lower than those observed for the Ponant and Vidourle lagoons that directly receive large rivers. Sampling stations were situated in lagoons (stations 1, 5, and 12), in channels connecting lagoons (stations 2, 3, 6, 8, and 11) or rivers (stations 4, and 10) to the sea, and on beaches (stations 7, and 9). Surface water (5 L) was collected in July and September 2011 at 12 stations, and in November 2011 at six stations. Temperature and salinity were recorded *in situ* using a WTW LF 196 conductimeter at the time of sampling. Samples were transported in coolers (15–18°C) to the laboratory and processed within 4 h after collection.

### *Vibrio* isolation

A Most Probable Number (MPN) method was applied to detect *V. parahaemolyticus*, and *V. cholerae* in water samples according Cantet et al. (2013). Enrichment of water samples (1 L, 100 and 10 mL filtered on 0.45 μm pore size nitrocellulose membranes and volumes of 1 mL, 0.1 and 0.01 mL) was performed in alkaline peptone water (APW). After incubation at 41.5°C for 18 h, 10 μl of each APW broth were spread onto CHROMagar™ Vibrio medium (Humeau, La Chapelle-sur-Erdre, France) and incubated at 37°C for 24 h. Typical colonies of *V. cholerae* and *V. parahaemolyticus* were selected for further identification.

### Molecular identification of *V. cholerae* and *V. parahaemolyticus* and MLST analysis

Prior to DNA analysis strains were grown overnight in APW at 37°C with shaking. Bacterial DNA was extracted from 1 mL of culture by heat lysis. Molecular identification of *V. cholerae* and *V. parahaemolyticus* were performed as previously described by PCR using specific primers for 16S–23S rRNA Intergenic Spacer Regions (ISR) (Chun et al., 1999) and *toxR* (Cantet et al., 2013), respectively.

The *ctxA* gene in *V. cholerae* and *tdh, trh1, trh2* genes in *V. parahaemolyticus* were detected as previously described by Hoshino et al. (1998) and Cantet et al. (2013), respectively. The genes included in the MLST schemes of *V. cholerae* (*adk, gyrB, mdh, metE, pntA, purM, pyrC*) and *V. parahaemolyticus* (*dnaE, gyrB, tnaA, recA, pyrC, pntA, dtdS*) were amplified as previously described by Octavia et al. (2013) and Gonzalez-Escalona et al. (2008), respectively.

PCR products and molecular weight marker (BenchTop 100 bp DNA Ladder, Promega) were separated in 1.5% (w/v) agarose gel in 0.5X Tris Borate EDTA (TBE) buffer. Amplification products were sequenced using forward primers on an ABI 3730xl automatic sequencer (LGC Genomics, Germany). Sequencing using reverse primer was performed when the sequences obtained with forward primers were not found in databases. The ssDNA sequences were deposited in the GenBank database with accession numbers from KJ656184 to KJ657569.

### Data analysis

#### Phylogeny

Gene sequences were codon-aligned using ClustalX after translation with TRANSLATE (http://www.expasy.org). The length of sequences used for further analyses is indicated in Table 1. Phylogenetic trees were reconstructed for each individual locus and for the 7 loci concatenated nucleotide sequences. Phylogenetic reconstructions were performed on IQ Tree server (Minh et al., 2013). The best-fit model was chosen for each dataset according to Akaike information criterion and used to reconstruct Maximum likelihood (ML) trees. Ultrafast bootstrap approximation approach (UFBoot) was used to compute the support of ML phylogenetic groups (Minh et al., 2013).

**Table 1:** **Sequence analysis of the seven loci studies for** ***Vibrio parahaemolyticus*** **and** ***Vibrio cholerae***.

	**Locus (length in pb)**	**Number of alleles**	**GC content**	**Number of polymorphic sites (%)**	**Number of non-synomynous codons**	**Genetic diversity (h)**	**dN ^a^**	**dS ^b^**	**dN/dS**
*Vibrio cholerae*	*adk* (416 bp)	27	0.485	27 (6.49%)	4	0.8862	0.0037	0.0267	0.1386
	gyrB (431 bp)	36	0.497	40 (9.28%)	1	0.9344	0.0031	0.0556	0.0558
	*mdh* (421 bp)	30	0.517	33 (7.84%)	0	0.9269	–	–	–
	*metE* (591 bp)	53	0.49	109 (18.44%)	16	0.9659	0.0041	0.1675	0.0245
	*pntA* (431 bp)	42	0.513	38 (8.82%)	4	0.9709	0.0034	0.0425	0.0800
	*purM* (476 bp)	18	0.531	62 (13.03%)	0	0.6984	–	–	–
	*pyrC* (449 bp)	50	0.504	105 (23.39%)	18	0.9743	0.0108	0.1854	0.0583
*Vibrio parahaemolyticus*	*dnaE* (557 bp)	53	0.486	127 (22.80%)	16	0.9837	0.0045	0.1173	0.0384
	*gyrB* (592 bp)	54	0.477	62 (10.47%)	4	0.9860	0.0088	0.1045	0.0842
	*tnaA* (423 bp)	34	0.488	27 (6.38%)	1	0.9308	0.0030	0.0430	0.0698
	*recA* (729 bp)	51	0.452	150 (20.58%)	16	0.9847	0.0034	0.1590	0.0214
	*pyrC* (493 bp)	49	0.483	41 (8.32%)	7	0.9747	0.0036	0.0511	0.0705
	*pntA* (430 bp)	35	0.438	29 (6.74%)	4	0.9512	0.0031	0.0389	0.0797
	*dtdS* (458 bp)	49	0.501	62 (13.54%)	3	0.9831	0.0101	0.4641	0.0218

a *dN, non-synonymous substitutions per non-synonymous site*.

b *dS, synonymous substitutions per synonymous site*.

#### Multi locus sequence typing (MLST) analysis

The alignment obtained for phylogenetic treeing was used for assignation of each isolate to a Sequence Type (ST) according to their allelic profiles and compared to accessible sequences on pubmlst databases (http://www.pubmlst.org). Allele profiles were also analyzed using goeBURST v3 software (http://goeburst.phyloviz.net/) to determine clonal complexes (CC) defined as sets of related strains that share at least five identical alleles on the 7 loci. The singleton (S) STs corresponded to STs differing from all the others by 3 or more of the 7 loci. The Phyloviz program employing eBURST algorithm was used to determine Minimum Spanning Tree that showed the relationships between STs (http://goeburst.phyloviz.net/). Dataset for each species are constituted of strains of this study and pubMLST isolates with complete MLST information: i.e., 228 isolates for *V. cholerae*, and 1513 isolates for *V. parahaemolyticus*.

#### Population genetics and recombination analyses

The number of polymorphic sites was calculated using DnaSP version 5.10 (http://www.ub.edu/dnasp/). The number of synonymous (dS) and non-synonymous (dN) substitutions per site was determined on codon-aligned sequences using SNAP software (http://www.hiv.lanl.gov/content/sequence/SNAP/SNAP.html). Program LIAN 3.5 (http://pubmlst.org/) was used to determine the h score of genetic diversity at each locus. The mean genetic diversity (H) was then calculated as the arithmetic mean of the h values for all loci. Rarefaction analysis was carried out using the freeware program aRarefactWin (http://www.uga.edu/strata/software/Software.html). Program LIAN 3.5 was also used to evaluate the recombination by calculation of the standardized index of association I_A_(sI_A_) and to test the null hypothesis of linkage disequilibrium. The homoplasy index φw test was done on pub MLST web site (http://www.pubmlst.org) or split tree. Recombination events were detected from aligned concatenated sequences using seven different methods with default parameters of the RDP v3.44 (Martin et al., 2010) software package: general, RDP, GENECONV, Bootscan, MAxChi, CHIMAERA, and Siscan. The r/m ratio, i.e., the relative impact of recombination and mutation in the diversification of the lineages, was calculated by LDhat (McVean et al., 2004) implemented in RDP v3.44. Recombination events were also visualized by decomposition analyses with SplitsTree 4.0 software using a distance matrix in nexus format generated from the concatenated sequence (http://www.splitstree.org/).

#### Statistics

Data were analyzed using R software available at http://www.R-project.org and R package FactoMineR (Lê et al., 2008). Distribution of data was determined by Shapiro-Wilk test. Wilcoxon test was used to determine differences between groups. ANOVA test was realized using R software and R package FactoMineR.

## Results

### Population structure and genetics of *V. cholerae* and *V. parahaemolyticus* in mediterranean coastal lagoons

During the three sampling campaigns (12 stations in July and September 2011 and 6 stations in November 2011) (Figure 1), 109 strains of non-toxigenic *V. cholerae*, i.e., negative in *ctxA*-specific PCR and 89 isolates of *V. parahaemolyticus* were isolated. All *V. parahaemolyticus* isolates were negative for *tdh, trh1* and *trh2* genes but one, strain 8.15, was positive for *trh2* gene only (Supplementary Tables S1, S2). The two *Vibrio* species were detected in all sites and approximately half of the strains were isolated from water collected in channels.

The 109 *V. cholerae* isolates displayed high genetic variability with 78 sequence types (STs) detected by MLST, the majority being represented by unique STs found only once and in one isolate (Supplementary Table S1). Only 14 STs (18%) were represented by multiple isolates. Three of them (ST255, 259, and 242) grouped strains isolated at different dates and sites, and in different salinity conditions. Seven other STs were each represented by isolates collected at the same date but in different sites (ST200, 201, 223, 246, 265, 270, 273). The 4 STs formed by strains isolated in a same site at the same date could probably be considered as replicates of a unique strain (ST 218, 219, 221, 226) (Supplementary Table S1). None of the STs described in this study except ST2 were documented in databases.

A similarly high genetic diversity was observed for *V. parahaemolyticus* isolates with 68 STs, 53 STs being represented by a single isolate and 15 STs (22.1%) by 2 to 5 isolates. All STs but two, represented by multiple isolates (*n* = 13), grouped strains originating from different sites, the most represented being ST481, ST2000 and ST2005 (Supplementary Table S2). Eleven of the 68 STs were previously described in databases.

**Figure 2:**
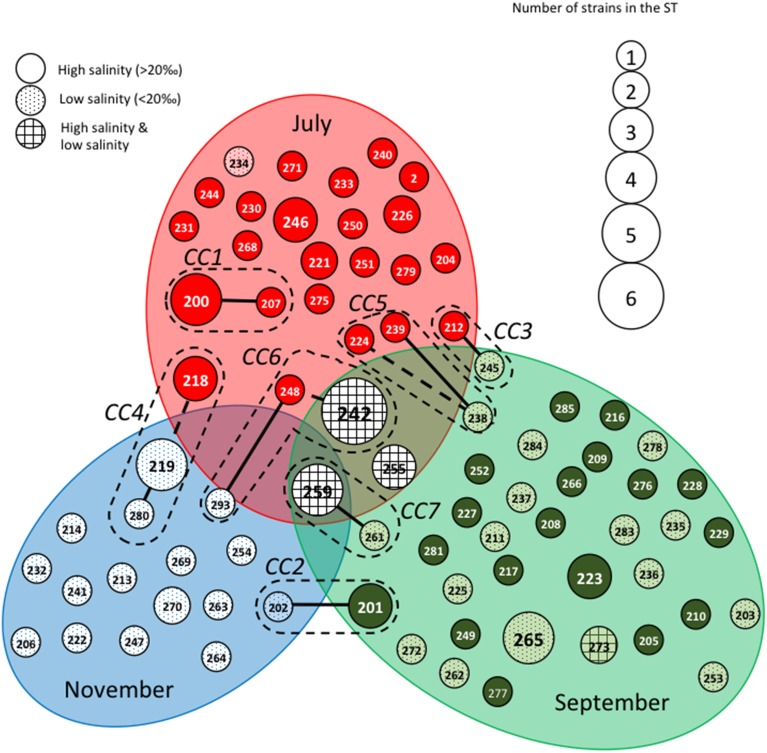
**Population structure of** ***Vibrio cholerae*** **based on MLST data after clustering by goeBURST software**. Each Sequence Type (ST) is represented by a circle with a size proportional to the number of strains by ST. STs that differed by 2 alleles (connected by dotted lines) or one (connected by full lines) are considered to belong to the same clonal complex (CC), which is surrounded by a dotted line. Gray ellipses group ST collected at the same date in July, September or November. The gray background of the circles representing the STs is related to the salinity at the time of sampling.

**Figure 3:**
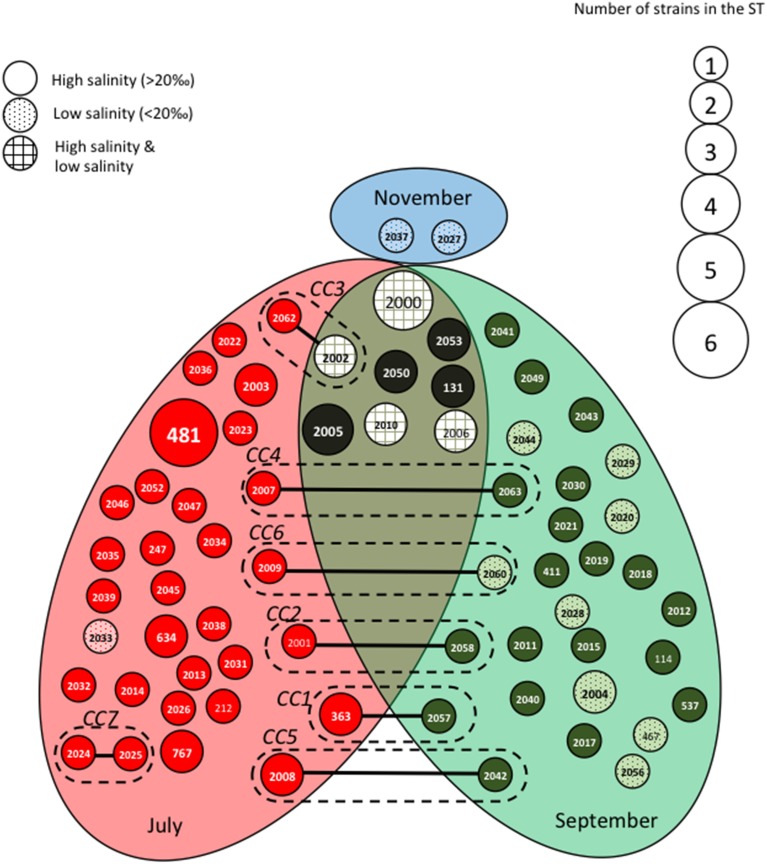
**Population structure of** ***Vibrio parahaemolyticus*** **based on MLST data after clustering by goeBURST software**. Each Sequence Type (ST) is represented by a circle with a size proportional to the number of strains by ST. STs that differed by one (connected by full lines) or two (connected by dotted lines) alleles are considered to belong to the same clonal complex (CC), which is surrounded by a dotted line. Gray ellipses group ST collected at the same date in July, September or November. The gray background of the circles representing the STs is related to the salinity at the time of sampling.

The STs clustering in clonal complexes (CCs) by goeBURST confirmed the high level of genetic diversity within the two species. The population of *V. cholerae* displayed seven small CCs containing 2 to 3 STs (Figure 2) (Supplementary Table S1). Seven CCs, each formed by only 2 STs, were also detected in the population of *V. parahaemolyticus* (Figure 3) (Supplementary Table S2).

The genetic diversity (H) was 0.9082 ± 0.0369 and 0.9706 ± 0.0081 for *V. cholerae* and *V. parahaemolyticus*, respectively. The genetic diversity at individual loci (h) and the number of single nucleotide polymorphism (SNP) are given in Table 1. For the two species, the rarefaction curve for each locus did not reach a plateau suggesting that we only partially explored the genetic diversity present in the ecosystem, thus confirming the high genetic diversity of the populations studied (Supplementary Figure S1).

In the two species, the level of polymorphic sites varied according to the genes from about 6 to 23% (Table 1). The multi-locus schemes used for *V. cholerae* and *V. parahaemolyticus* shared 3 identical loci, *gyrB, pntA*, and *pyrC*. The levels of polymorphism of *gyrB* and *pntA* were similar in the two species whereas *pyrC* displayed a much higher polymorphism in *V. cholerae* than in *V. parahaemolyticus*. In the two species, the numbers of SNPs at the 7 loci were close, 414 (12.9%) for *V. cholerae* and 498 (13.5%) for *V. parahaemolyticus*. None of these SNPs produced premature stop codon. The frequency of non-synonymous mutations was low for each locus in both species, the highest dN/dS ratio being 0.1386 for the gene *adk* in *V. cholerae* (Table 1).

Altogether, genetic data from *V. cholerae* and *V. parahaemolyticus* multi-locus schemes showed high genetic diversity in genes subjected to low selective pressure.

### Genetic diversity of *V. cholerae* and *V. parahaemolyticus* from mediterranean lagoons compared to worldwide population

We compared the multi-locus genetic data of the *Vibrio* population studied herein with those available in pubMLST databases for clinical and environmental isolates originating from various countries and collected at different times. Reconstruction of minimum-spanning trees from MLST profiles obtained in pubMLST databases and in this study showed no specificity in the distribution of the strains from Mediterranean coastal lagoons. They were equally distributed in the different clusters and globally reflected the overall diversity of *V. cholerae* and *V. parahaemolyticus* strains available in pubMLST databases (data not shown). H and ST/strain scores were similar in a collection sampled during 6 months in Mediterranean lagoons and in *Vibrio* collections from databases. The H score was 0.8836 in database vs. 0.9082 in this study for *V. cholerae* and H was 0.9536 in database vs. 0.9705 in this study for *V. parahaemolyticus*. Therefore, the very high genetic diversity observed locally in the coastal lagoon systems for the two *Vibrio* species was similar to that observed in strains collected from many countries (five continents) over the past 60 years (data not shown).

Among the 78 STs detected in this study for *V. cholerae* population, ST2 was the only ST previously deposited in the database. It corresponded to *ctx*-strains of serotype O49 isolated in Bangladesh in 2000 in a context of sporadic digestive infections (Octavia et al., 2013). It belonged to the same CC as ST1 (serotype O49) previously isolated in the same epidemiological context. ST223 and ST238 belonged to CCs that grouped clinical strains of *V. cholerae*. ST223 was related to ST65 corresponding to O37 strains isolated in the environment in Germany in 1993 and in sporadic digestive infections in Bangladesh in 2000. ST238 was related to ST30 isolated in human in Peru in 1994.

For *V. parahaemolyticus* population, 11 STs among the 68 detected herein were previously deposited in pubMLST database, which is larger than the *V. cholerae* database. Most of them corresponded to strains isolated from water or shellfish, worldwide (USA, China, Thailand, Vietnam and Northern Europe) from 1997 to 2012. They presented the virulence genotype *tdh*-/*trh*-, except for one environmental strain isolated in USA (Pacific coast) in 2006 that was *tdh*+/*trh*+. The ST363 that contained 2 strains isolated in sites 5 and 10 in July 2011 was composed of clinical strains (O3, *tdh*+/*trh*+) isolated in a patient with gastro-enteritis in Thailand in 1991. The ST3, which represented 15% of the strains deposited in MLST database and corresponded to the pandemic virulent strain O3:K6, was not detected in this study.

### Genetic diversity of *V. cholerae* and *V. parahaemolyticus* according to stations, times of sampling and salinity

The 109 isolates of *V. cholerae* were equally distributed whatever the month of sampling, the station or the salinity (Supplementary Table S3) while *V. parahaemolyticus* population was not, particularly according to salinity conditions. Indeed, *V. parahaemolyticus* was distributed in two groups, a high salinity group composed of 73 isolates and a low salinity group of 16 isolates (Supplementary Table S4).

Genetic data of strains of the two species were compared according to the month of sampling (July, September, November 2011), the sampling stations (lagoon, beach, channel between river and sea, and channel between lagoon and sea) and water salinity (high, i.e., > 20 and low, i.e., < 20) (Supplementary Table S5). For *V. cholerae*, the diversity assessed by the ST/strain score showed significant differences according to salinity, with the number of ST/strain significantly higher (*p* < 0.005) when the salinity was low. For *V. parahaemolyticus*, the ST/strain score was higher in September 2011 (0.95) than in July 2011 (0.795) but the difference was not significant (*p* = 0.41) (Supplementary Table S4). However, as observed for *V. cholerae*, the ST/strain score for *V. parahaemolyticus* was significantly higher (*p* < 0.005) when the salinity was low. Both species' genetic diversity was also significantly higher in seawater from beach stations than in samples from other sampling stations (*p* < 0.005) but this could be due to a sampling bias because of the low number of strains collected from seawater.

Figures 2, 3 show the qualitative distribution of STs by goeBURST according to sampling dates and salinity. For the two species, clustering by reconstruction of minimum spanning tree gave the same results as goeBURST clustering (data not shown). A large majority of *V. cholerae* STs and strains was represented at only one date of sampling. Indeed, only one ST (ST259), containing five isolates, was found during the three campaigns in four different sites while two others (ST242 and ST255) were found in July and September 2011 (Figure 2). As expected, high salinities were recorded in July and low salinities in November whereas the high and low salinities observed in September were dependent on the sampling stations. Most STs (all but ST273) found only in September were detected either at high or low salinity whereas only a few STs found in July and September (ST242, ST255, and ST259) were detected in both salinity conditions. The STs forming CCs were mostly distributed among two or three sampling campaigns, except for CC1 that contained only isolates harvested in July. Most CCs contained STs detected in high salinity samples and closely related STs detected in low salinity samples (Figure 2). They also contained the same or related STs detected in different stations (Supplementary Figure S2). This suggested that slight variations in the genotypes could be related to changes in environmental conditions.

When matched with the stations, the types of station and the watersheds feeding the station (Supplementary Figure S2), the population structure and distribution of STs appeared totally independent from the sampling stations. Most STs that grouped several isolates were composed of strains isolated from different stations, different types of station and different watersheds. For instance, isolates from channels and lagoons could share the same ST and/or CC and so could isolates from channels and seawater.

We observed roughly the same results for *V. parahaemolyticus* with July and September populations seldom overlapping (Figure 3). Among the eight STs shared between July and September, four were detected in both high and low salinities. As observed for *V. cholerae*, all CCs but one (CC7) grouped STs detected in different sampling campaigns but mostly at similar low salinities (Figure 3). Again, the distribution of STs and CCs appeared totally independent from the sampling stations (Supplementary Figure S3).

### Phylogeny of *V. cholerae* and *V. parahaemolyticus*

**Figure 4:**
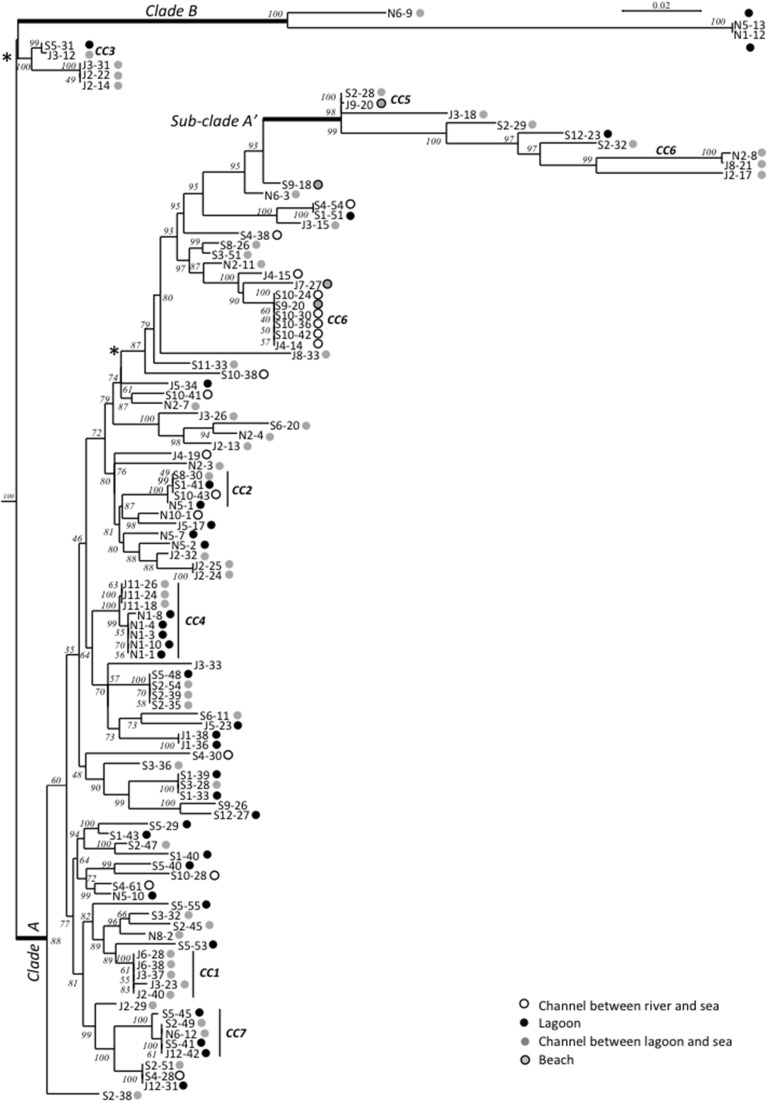
**Maximum-Likelihood tree based on concatenated sequences of the seven housekeeping gene fragments of the MSLT scheme indicating the relative placement of** ***V. cholerae*** **isolates**. The numbers at the nodes are bootstrap values in %. *V. mimicus* ATCC 33653 was used as the out-group organism. Circles beside the isolate names correspond to the type of sampling sites as given in the color legend. The scale bar indicates the number of substitutions per nucleotide position. ^*^branches submitted to long branches attraction artifact caused by sub-clade A' and B. When the tree was reconstructed without sub-clade A' and clade B sequences, the branches marked with asterisks took place inside the clade A without branching particularities.

**Figure 5:**
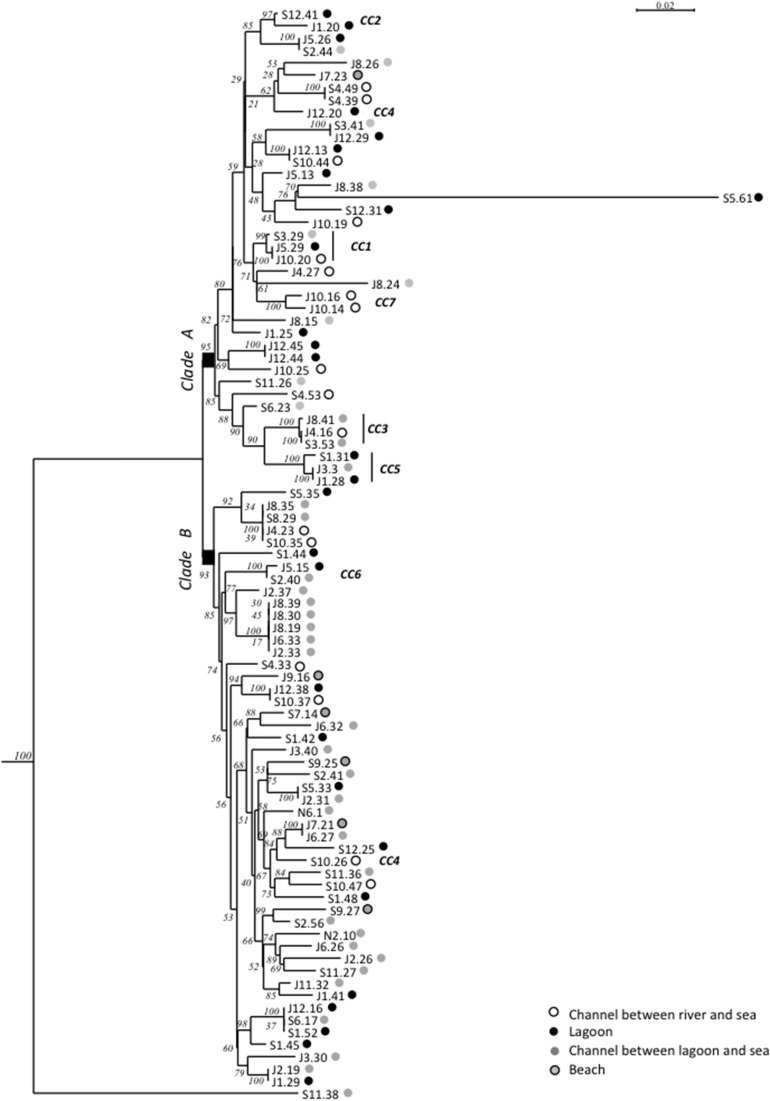
**Maximum-Likelihood tree based on concatenated sequences of the seven housekeeping gene fragments of the MSLT scheme indicating the relative placement of** ***V. parahaemolyticus*** **isolates**. The numbers at the nodes are bootstrap values in %. *V. azureus* NBRC 104587 was used as the out-group organism. Circles beside the isolate names correspond to the type of sampling sites as given in the color legend. The scale bar indicates the number of substitutions per nucleotide position.

Maximum-Likelihood phylogenies based on concatenated sequences of the seven housekeeping gene fragments of the MSLT scheme (3215 bp for *V. cholerae* and 3682 bp for *V. parahaemolyticus*) are shown in Figures 4, 5. Major phylogenetic clusters were congruent with the CCs determined by goeBURST with the exception of *V. cholerae* CC6 and *V. parahaemolyticus* CC4, which split into distinct parts in ML phylogeny. As observed above with goeBURST structuration, phylogeny showed only a few sporadic clusters of *V. cholerae* and *V. parahaemolyticus* composed by a few strains from a same date or a same station (Figure 4 and Supplementary Figure S2 part B).

The *V. cholerae* ML tree (Figure 4) displayed 3 main clades. The main one (clade A) included the majority of isolates and STs and inside this clade, a sub-clade named A′ displayed a higher level of genetic diversity with emerging long branches. Clade B was atypical due to an external position and long branches. In monogenic-based trees (data not shown), clade B isolates were either in the *V. cholerae* clade (*adk, mdh, pntA*, and *pyrC*-based trees) or in *V. metoecus* and *Vibrio parilis* clades (*gyrB* and *purM*-based trees) remote from *V. cholerae* or on an independent lineage (*metE*-based tree). Comparison of *metE* and *purM* gene sequences of strains N1-12 and N5-13 with sequences of databases showed a best match of only 93% with other published sequences of *Vibrio* spp. The *gyrB* gene of N1-12 and N5-13 gave the best match with strains *Vibrio* sp. W0706-81 (HM009674), L8M (HM009590), and HB0308-2A5 (HM009568) non-affiliated to the species level and detected in coastal marine water in California and Hawaii. The third member of clade B, N6-9, was generally related to N1-12 and N5-13, except for *purM* phylogeny that placed it on a long independent branch remote from all other *Vibrio* spp. *purM* sequences, and for *metE* phylogeny that placed it in the *V. cholerae* clade.

**Figure 6:**
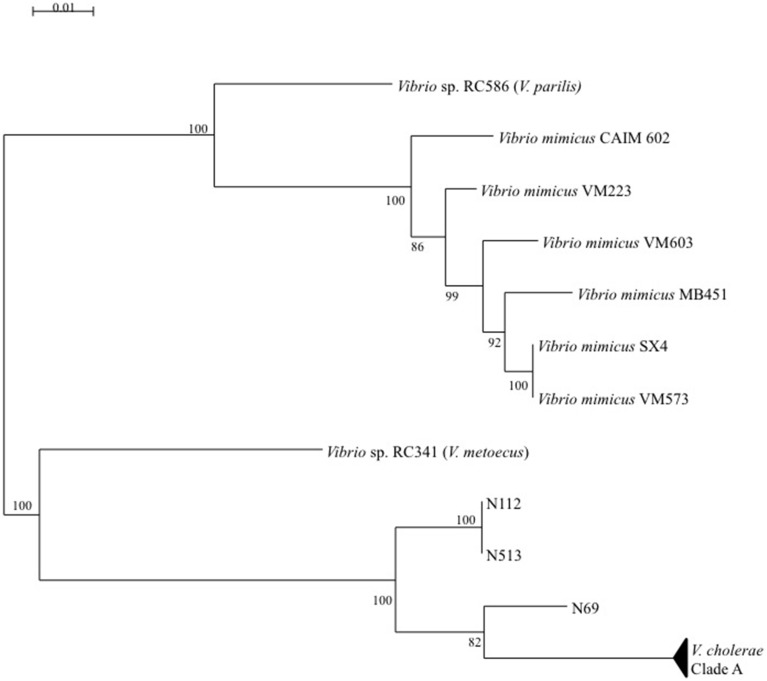
**Maximum-Likelihood tree based on concatenated sequences of the seven housekeeping gene fragments of the MSLT scheme indicating the relative placement of** ***V. cholerae*** **clade B isolates regarding** ***V. metoecus*****,** ***V. mimicus*****, and** ***V. parilis***. The scale bar indicates the number of substitutions per nucleotide position.

Multi-locus and mono-locus phylogenies showed that clade B could not be affiliated with certainty to *V. cholerae* in spite of the positive *V. cholerae* 16S–23S rRNA ISR PCR for isolates N1-12, N5-13, and N6-9 (Figure 6). These conflictual phylogenies suggested that Horizontal Gene Transfer (HGT) was involved in clade B emergence. HGT of *pyrC* gene among *V. cholerae, V. metoecus, V. mimicus*, and *V. parilis* was also suspected for most strains of the sub-clade A′.

Two main clades were shown in the *V. parahaemolyticus* tree (Figure 5). The structure of the tree appeared independent from dates and sampling stations. The long branch observed for isolate S5.61 was caused by a very atypical *dnaE* sequence, which displayed less than 84% similarity with other members of the species but 98.7% identity with *V. mimicus* ATCC 33655 *dnaE* sequence. Other best matches were *V. cholerae* strain 7449 (98.5%), and *Vibrio* sp. HB0308 and L8M (96.3%), which were previously found related to *V. cholerae* clade B strains (N5-13, N1-12) in *gyrB* phylogeny. This result indicated genetic exchanges between *Vibrio* sp. related to *V. cholerae* and *V. parahaemolyticus*. It is noteworthy that *Vibrio* sp. N5-13 and *V. parahaemolyticus* S5.61 were isolated in the same station in November and September, respectively. *V. parahaemolyticus* J8.24 and S11.38 were also placed on long branches due to very polymorphic *dtdS* and *gyrB* alleles, respectively. Comparison to databases showed that the best matches of isolate J8.24 for *dtdS* was 94% with *Vibrio campbelli* and *Vibrio harveyi* type strains. The *recA* gene of strain S11.38 displayed 98.8% identity with *V. mimicus* strain LMG 7896^T^ and only 86% with the type strain of *V. parahaemolyticus* LMG2850^T^. Therefore, we described a genetic exchange occurring between two species of pathogenic vibrios, *V. parahaemolyticus* and *V. mimicus* but also between *V. parahaemolyticus* and particular genotypes related to *V. cholerae*.

### Genetic recombination in *Vibrio* populations of mediterranean coastal lagoon systems

**Figure 7:**
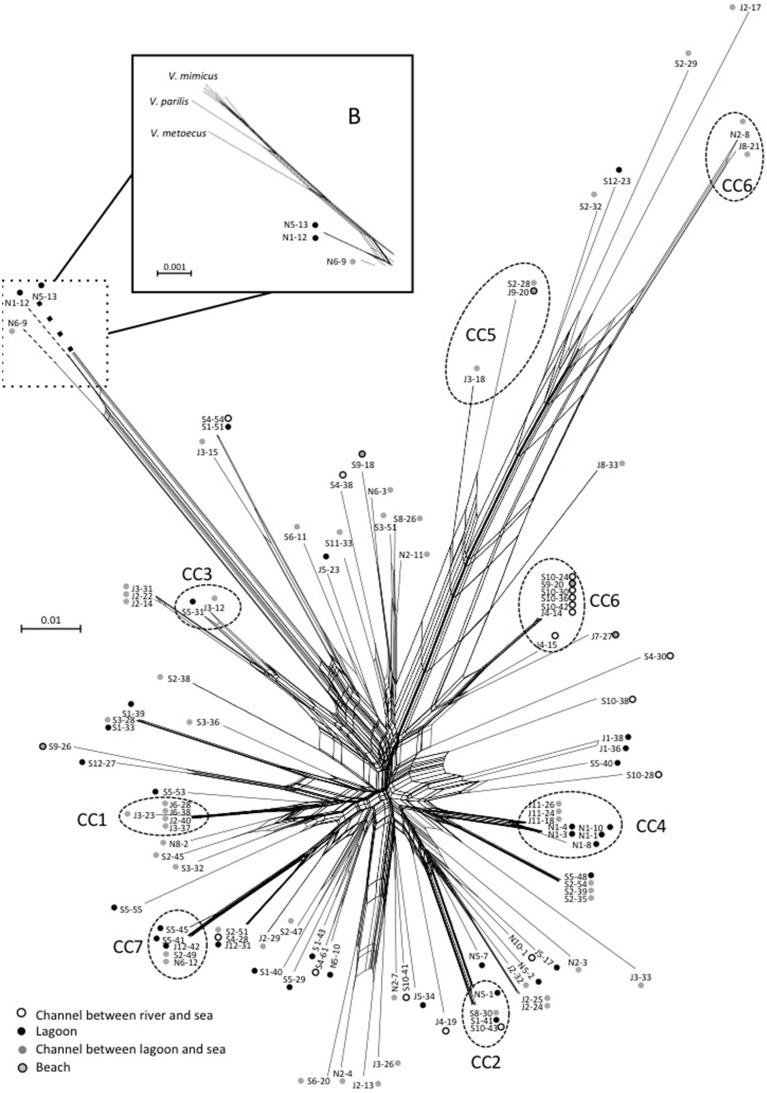
**Neighbor-net graph reconstructed from the concatenated sequences of the 109 strains of** ***V. cholerae*** **using Splits Tree 4.0**. A network-like graph indicates recombination events. ST numbering and type of sampling site (as indicated in the legend) are shown at the branch tips. The position of *V. metoecus, V. parilis*, and *V. mimicus* regarding the emerging sub-clade B is indicated in the frame B. Dotted circles indicated clonal complexes as determined by goeBURST. The scale bars indicate the number of substitutions per nucleotide position.

**Figure 8:**
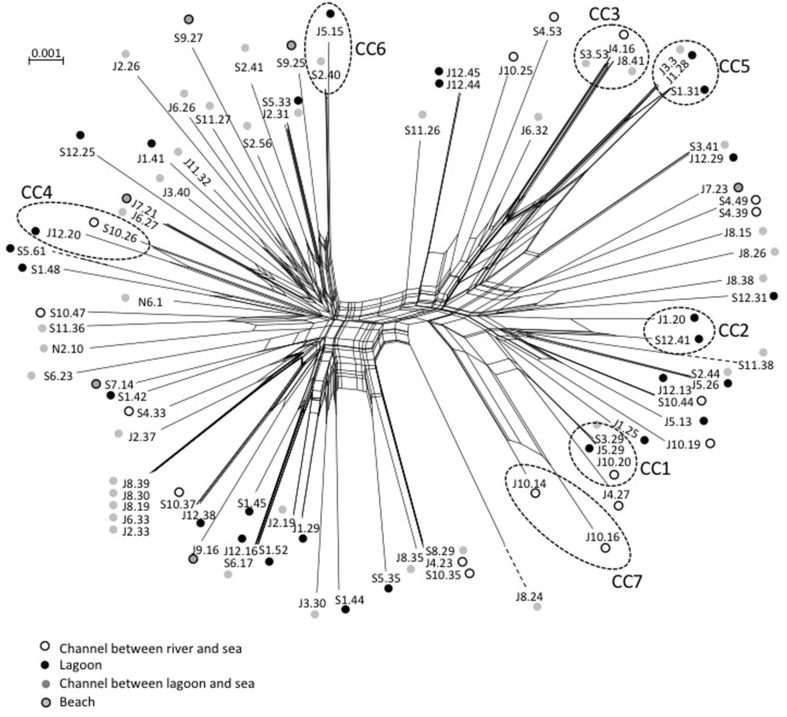
**Neighbor-net graph reconstructed from the concatenated sequences of the 89 strains of** ***V. parahaemolyticus*** **using Splits Tree 4.0**. A network-like graph indicates recombination events. ST numbering and type of sampling site (as indicated in the legend) are shown at the branch tips. Dotted circles indicated clonal complexes as determined by goeBURST. The scale bars indicate the number of substitutions per nucleotide position.

Events of recombination by HGT showed by phylogenetic incongruences were also supported by decomposition analysis using Neighbor-Net reconstruction, which revealed interconnected networks for both *V. cholerae* (Figure 7) and *V. parahaemolyticus* (Figure 8). Recombination events occurred in the CCs, among CCs, among singletons but also between CCs and singletons (Figures 7, 8). The two main clades detected in *V. parahaemolyticus* ML phylogeny were also seen by decomposition analysis showing that recombination events occurred more frequently inside each clade than between clades (Figure 8). Most CCs observed in the two species involved branches with recombining signals. In *V. cholerae*, major events of recombination led to the emergence of CC5, CC6 and clade B (Figure 7). These emerging branches were similar to the branches corresponding to the speciation of *V. mimicus, V. metoecus*, and *V. parilis* (Figure 7). These results suggested that recombination was a major force driving diversification and speciation of vibrios in Mediterranean coastal lagoon systems.

Recombination was then evaluated in *V. cholerae* and *V. parahaemolyticus* by the determination of standardized index of association (sI_A_), the homoplasy index φw test and by using RDP software. The sI_A_, expected to be null when a population is at linkage equilibrium, was significantly different from zero for both *V. cholerae* (sI_A_ = 0.1697; *p* = 0.0002) and *V. parahaemolyticus* (sI_*A*_ = 0.3307; *p* < 10^3^). No significant sI_A_ difference was observed according to dates, stations, and salinities for both species (data not shown). The sI_A_ values indicated significant linkage disequilibrium suggesting that *V. cholerae* and *V. parahaemolyticus* population conserved a clonal backbone despite frequent events of recombination. In contrast, the homoplasy index φw test, which discriminates between recurrent mutation and recombination, found statistically significant evidence for recombination (*p* = 0.0) in both *V. cholerae* and *V. parahaemolyticus* populations.

Recombination events within these two *Vibrio* populations were further analyzed by RDP software. In *V. cholerae* population detected by at least 4 out of the 7 methods performed by RDP, 34 strains (31.2% of isolates) underwent at least one recombination event (Supplementary Table S6). Recombination events involved *metE, pyrC*, and *pntA* genes. For *V. cholerae*, the sampling month and the salinity influenced slightly the frequency of recombining isolates, a low salinity being associated with a lower frequency of recombining isolates. In *V. parahaemolyticus* population, 52 strains (58.4%) underwent at least one recombination event (Supplementary Table S7). The gene *recA* was involved in recombination events for 56.1% of the strains. No particular environmental condition influenced the frequency of recombination.

Finally, the relative impact of recombination and mutation in the diversification of the lineages was calculated. The r/m ratio was 37 and 12 for *V. cholerae* and *V. parahaemolyticus*, respectively. These values indicate a very high impact of recombination as defined by Vos and Didelot (2009).

## Discussion

The outstanding clinical and epidemic manifestations of cholera have led to consider *V. cholerae* separately from other potentially pathogen vibrios. However, *V. cholerae* and *V. parahaemolyticus* share environmental niches and display similar lifestyles, as halophilic bacteria that occasionally infect humans. Previous data showed that both species presented a high genetic diversity with a clonal structure on a background of recombination, i.e., a classical epidemic structure (Luo et al., 2013). The ecological and population structure similarities between *V. cholerae* and *V. parahaemolyticus* prompted us to compare the two species living in the same ecosystem. Herein, we explored the genetic structure of *V. cholerae* and *V. parahaemolyticus* populations collected together in a cholera-free region without any recent history of major outbreaks of human vibriosis. The presence of non-O1/non-O139 *V. cholerae* and *V. parahaemolyticus* had been previously demonstrated in a French coastal region (Deter et al., 2010; Cantet et al., 2013). In this study, the sampling and analysis of waters from 12 different sites in a small region of about 30 km^2^ showed the very high degree of genetic diversity of *V. cholerae* and *V. parahaemolyticus* in Mediterranean coastal lagoon systems.

The isolates harvested in Mediterranean lagoons did not carry any major virulence factors, except for one isolate of *V. parahaemolyticus* that carried only the *trh2* gene. The frequency of virulence factors in environmental populations of *V. parahaemolyticus* (1.1%) and *V. cholerae* (0%) was lower, especially for *V. parahaemolyticus*, than that observed in waters collected from other French coastal areas (English Channel, Atlantic and Mediterranean coasts)(Hervio-Heath et al., 2002; Deter et al., 2010). Studies in cholera-free temperate or cold regions are scarce but all have underlined the absence of *ctx*+ isolates in environmental strains (Zo et al., 2009; Schuster et al., 2011; Haley et al., 2012) whereas toxigenic strains were mainly detected in outbreak contexts (Tobin-D'Angelo et al., 2008; Haley et al., 2014).

Databases comparison showed that most STs described in this study were detected for the first time, demonstrating that the *V. cholerae* and *V. parahaemolyticus* populations are extremely diverse and their genetic diversity under-reported. In this study, the distribution of STs, as well as the H and ST/strain scores, are in agreement with the overall diversity of *V. cholerae* and *V. parahaemolyticus*. Indeed, we observed locally a genetic diversity similar to or higher than that observed worldwide in the last fifty years. This has been previously shown for *V. parahaemolyticus* populations (Gonzalez-Escalona et al., 2008; Yan et al., 2011) and for *V. cholerae* populations. Zhang et al. (2014) demonstrated that non-toxigenic isolates exhibited a greater diversity than did their toxigenic counterparts, suggesting that the acquisition of virulence factors and pathogenic behavior changes the population genetics of vibrios, probably by clonal selection and success during human infectious outbreaks.

The vibrios diversity appeared independent from the type of sampling site and the type of lagoon system. Moreover, we did not detect any clones specialized locally according to a particular site in the lagoon system, but this could be due to the hydrodynamic conditions in these systems where water is extensively mixed. Some STs detected in Mediterranean lagoon systems were previously detected elsewhere in the world, in very different environmental conditions or in clinical samples. This suggests the absence of geographic or lifestyle specialization of *V. cholerae* and *V. parahaemolyticus* populations collected in cholera-free or vibriosis outbreak-free regions. Moreover, some STs without virulence factors identified in this study had been previously detected as virulent strains, suggesting that the population described here could provide the genetic background for the emergence of pathogenic clones (Li et al., 2014).

To our knowledge, the study of Ellis et al. (2012) and the present study are the only ones considering the genetic diversity of *V. parahaemolyticus* according to local variation of environmental conditions. The level of genetic diversity of *V. cholerae* and *V. parahaemolyticus* was higher when the salinity was low. Some of *V. parahaemolyticus* CCs detected in both low and high salinity conditions are composed of closely related STs, each being detected in only one condition of salinity. Similarly, *V. cholerae* CCs detected in different sites are formed by closely related STs. These slight differences in nucleotide sequences suggest microevolution events spurred by adaptation to changing environmental conditions. In New Hampshire shellfish waters, the diversity in *V. parahaemolyticus* populations increased as water temperature increased (Ellis et al., 2012). The increased diversity associated with warming waters and low salinity associated with precipitations suggests again that new pathogenic lineages may emerge with climate change.

It is well-known that the emergence of pathogenic clones in vibrios is linked to HGT of virulence genes or genetic elements among strains as for phage CTX in *V. cholerae* (Hazen et al., 2010). Several factors have been shown to influence the recombination rate in vibrios. In *V. cholerae*, high chitin concentrations induce an increase of recombination rate (Meibom et al., 2005). Moreover, SOS system activation by environmental factors stimulates integrase activity that in turn increases the frequency of transformation (Guerin et al., 2009). We studied the recombination rate according to the site and salinity, and showed that recombination events were more frequent in high-salinity conditions for *V. cholerae*. This could be related to the positive association between salinity and the rate of HGT detected by genomics (Jain et al., 2003). More generally, we describe a high rate of recombination in *V. parahaemolyticus* and *V. cholerae* populations. For some of the recombining STs, the donor STs were detected in the Mediterranean lagoon ecosystems. This demonstrates that, beside SNPs described above as related to changes in environmental conditions, recombination is a diversity-generating mechanism ongoing in Mediterranean lagoons. This has been previously described for environmental strains but not for clinical strains of *V. parahaemolyticus* (Chowdhury et al., 2004a; Ellis et al., 2012), which is consistent with an epidemic spread of a subset of virulent clones. Similarly, previous studies on disease-causing strains have shown *V. cholerae* to be a primarily clonal bacterial species, but the vast diversity in environmental strains showed a recombination rate of 6.5 times the mutation rate (Keymer and Boehm, 2011). One could hypothesize that environmental *Vibrio* spp. populations form a reservoir where the diversity is efficiently enhanced by recombination in order to produce a generalist population from which specialized clones can emerge. Moreover, we showed that HGT of housekeeping genes occurred among species of potentially pathogenic vibrios in Mediterranean coastal systems. In multilocus phylogeny, such HGT leads to emerging branches similar to those supporting newly described vibrios species, *V. mimicus* (Davis et al., 1981), and *V. metoecus* (Kirchberger et al., 2014). This suggests that recombination events such as the ones we describe in Mediterranean coastal systems may drive speciation among potentially pathogenic vibrios.

## Conclusive remarks

Mediterranean coastal lagoons host *V. cholerae* and *V. parahaemolyticus* showing a genetic diversity equivalent to the worldwide diversity described so far. The current very low frequency of virulence genes in these populations indicates a low risk for human infection. But the presence of STs involved in human infections elsewhere, as well as the frequent recombinations observed in these transitional aquatic ecosystems, indicates that autochthonous *V. cholerae* and *V. parahaemolyticus* populations could be involved in epidemiological cycles and infectious hazard.

### Conflict of interest statement

The authors declare that the research was conducted in the absence of any commercial or financial relationships that could be construed as a potential conflict of interest.
