# Supplementary material for: Highly diverse recombining populations of Vibrio cholerae and Vibrio parahaemolyticus in French Mediterranean coastal lagoons
Source: Front Microbiol. 2015 Jul 16;6:708. doi: 10.3389/fmicb.2015.00708 (PMC4503927; doi:10.3389/fmicb.2015.00708)

***Supplementary Material***

**Highly diverse recombining populations of *Vibrio cholerae* and *Vibrio parahaemolyticus* in French Mediterranean coastal lagoons**

Kévin Esteves^1^, Thomas Mosser^1^, Fabien Aujoulat^1^, Dominique Hervio-Heath^2^, Patrick Monfort^1^, Estelle Jumas-Bilak^1,3*^

^1^Team “Pathogènes Hydriques Santé, Environnements”, HydroSciences Montpellier, UMR 5569, Université de Montpellier, CNRS, IRD, France

^2^Laboratoire Santé, Environnement et Microbiologie, Ifremer, RBE, SG2M, Plouzané, France

^3^Département d’Hygiène Hospitalière, Centre Hospitalier Universitaire, Montpellier, France

**Corresponding author:**

Pr Estelle Jumas-Bilak, "HydroSciences", UMR 5569, Equipe "Pathogènes Hydriques Santé Environnement", Faculté de Pharmacie , 15, Avenue Charles Flahault, BP 14491, 34093 Montpellier Cedex 5, France, ebilak@univ-montp1.fr

**Supplementary Figure S1**

A

B

**Supplementary Figure S1**: Genetic profile accumulation chart generated as rarefaction curves on the site Online Calculation (http://fastgroup.sdsu.edu/cal_tools.htm). The curves show the evolution of the number of unique profiles detected during analysis for each or all alleles and for *V. cholerae* (A) and *V. parahaemolyticus* (B) populations.

**Supplementary Figure S2**


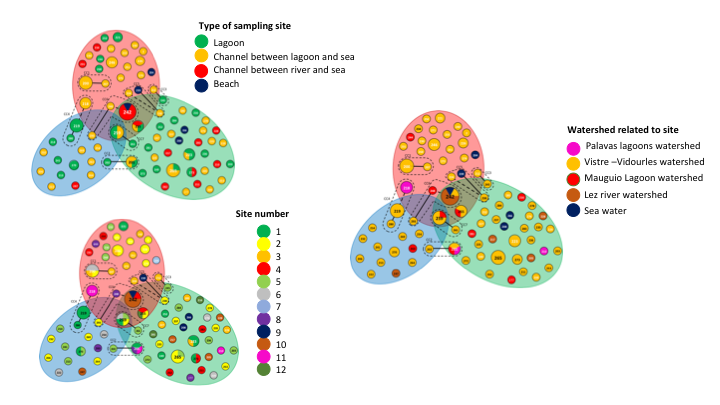


**Supplementary Figure S2:** Population structure of *Vibrio cholerae* based on MLST data after clustering by goeBURST software. Each Sequence Type (ST) is represented by a circle with a size proportional to the number of strains by ST as presented in Figure 2. STs that differed by 2 alleles or less are considered to belong to the same clonal complex (CC), which is surrounded by a dotted line. Grey ellipses group ST harvested at the same date in July, September or November 2011. The colour of the circles representing the STs were related to the site of sampling, to the type of site of sampling and to the watershed related to the site of sampling, as presented in the color legends.

**Supplementary Figure S3**


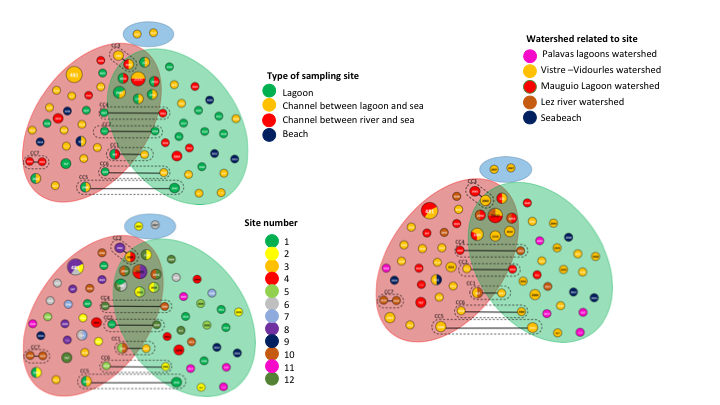


**Supplementary Figure S3:** Population structure of *Vibrio parahaemolyticus* based on MLST data after clustering by goeBURST software. Each Sequence Type (ST) is represented by a circle with a size proportional to the number of strains by ST as presented in Figure 3. STs that differed by 2 alleles or less are considered to belong to the same clonal complex (CC), which is surrounded by a dotted line. Grey ellipses group ST harvested at the same date in July, September or November 2011. The colour of the circles representing the STs were related to the site of sampling, to the type of site of sampling and to the watershed related to the site of sampling, as presented in the color legends.

## Supplementary Tables

The following tables gather all the raw data used for the analyses and figures presented in the manuscript. Data are presented for each strain (Tables S1 and S2) and for each locus (Tables S3 and S4). Table S5 presents comparative results of Wilcoxon and ANOVA statistics. Tables S6 and S7 present the recombination events detected in the 2 populations of *V. cholerae* and *V. parahaemolyticus*.

**Supplementary Table S1:** Date of sampling, origin, water salinity during sampling and multi-locus genotype for *Vibrio cholerae* strains analyzed in this study.

| **Date** | **Site** | **Salinity**  **(**‰**)** | **Isolate** | **Sequence**  **type (ST)** | ***adk***  **type** | ***gyrB***  **type** | ***mdh***  **type** | ***metE***  **type** | ***pntA***  **type** | ***purM***  **type** | ***pyrC***  **type** | **Clonal**  **Complex**  **(CC)** |
| --- | --- | --- | --- | --- | --- | --- | --- | --- | --- | --- | --- | --- |
| July | 1 | 28.4 | J136 | 221 | 2 | 108 | 21 | 112 | 107 | 1 | 108 |  |
|  |  |  | J138 | 221 | 2 | 108 | 21 | 112 | 107 | 1 | 108 |  |
|  | 2 | 23 | J213 | 271 | 109 | 55 | 14 | 64 | 38 | 107 | 107 |  |
|  |  |  | J214 | 246 | 14 | 101 | 108 | 106 | 108 | 101 | 109 |  |
|  |  |  | J217 | 2 | 1 | 1 | 4 | 48 | 29 | 17 | 41 |  |
|  |  |  | J222 | 246 | 14 | 101 | 108 | 106 | 108 | 101 | 109 |  |
|  |  |  | J224 | 226 | 6 | 23 | 53 | 47 | 11 | 1 | 114 |  |
|  |  |  | J225 | 226 | 6 | 23 | 53 | 47 | 11 | 1 | 114 |  |
|  |  |  | J229 | 279 | 116 | 105 | 15 | 100 | 111 | 100 | 102 |  |
|  |  |  | J232 | 233 | 13 | 36 | 52 | 47 | 117 | 1 | 141 |  |
|  |  |  | J240 | 200 | 1 | 5 | 14 | 142 | 38 | 1 | 100 | CC1 |
|  | 3 | 30.3 | J312 | 212 | 1 | 101 | 14 | 69 | 100 | 1 | 103 | CC3 |
|  |  |  | J315 | 240 | 14 | 14 | 4 | 105 | 22 | 17 | 35 |  |
|  |  |  | J318 | 224 | 6 | 6 | 29 | 11 | 25 | 1 | 3 | CC5 |
|  |  |  | J323 | 207 | 1 | 5 | 14 | 142 | 38 | 101 | 100 | CC1 |
|  |  |  | J326 | 251 | 14 | 50 | 14 | 120 | 123 | 1 | 107 |  |
|  |  |  | J331 | 246 | 14 | 101 | 108 | 106 | 108 | 101 | 109 |  |
|  |  |  | J333 | 275 | 50 | 5 | 115 | 145 | 122 | 1 | 140 |  |
|  |  |  | J337 | 200 | 1 | 5 | 14 | 142 | 38 | 1 | 100 | CC1 |
|  | 4 | 15.4 | J414 | 242 | 14 | 36 | 46 | 36 | 57 | 1 | 111 | CC6 |
|  |  |  | J415 | 231 | 13 | 1 | 2 | 36 | 65 | 1 | 112 |  |
|  |  |  | J419 | 234 | 13 | 44 | 14 | 128 | 3 | 9 | 39 |  |
|  | 5 | 30.2 | J517 | 230 | 7 | 41 | 15 | 47 | 107 | 100 | 47 |  |
|  |  |  | J523 | 250 | 14 | 114 | 124 | 123 | 101 | 101 | 109 |  |
|  |  |  | J534 | 268 | 107 | 5 | 14 | 118 | 47 | 1 | 117 |  |
|  | 6 | 34.4 | J628 | 200 | 1 | 5 | 14 | 142 | 38 | 1 | 100 | CC1 |
|  |  |  | J638 | 200 | 1 | 5 | 14 | 142 | 38 | 1 | 100 | CC1 |
|  | 7 | 36.5 | J727 | 204 | 1 | 4 | 14 | 35 | 120 | 5 | 46 |  |
|  | 8 | 29.3 | J821 | 248 | 14 | 49 | 46 | 36 | 57 | 1 | 20 | CC6 |
|  |  |  | J833 | 244 | 14 | 36 | 100 | 132 | 101 | 111 | 143 |  |
|  | 9 | 36.3 | J920 | 239 | 14 | 6 | 29 | 87 | 25 | 1 | 124 | CC5 |
|  | 11 | 36.5 | J1118 | 218 | 2 | 5 | 15 | 9 | 54 | 1 | 35 | CC4 |
|  |  |  | J1124 | 218 | 2 | 5 | 15 | 9 | 54 | 1 | 35 | CC4 |
|  |  |  | J1126 | 218 | 2 | 5 | 15 | 9 | 54 | 1 | 35 | CC4 |
|  | 12 | 23.3 | J1231 | 255 | 101 | 36 | 102 | 100 | 64 | 100 | 108 |  |
|  |  |  | J1242 | 259 | 101 | 41 | 103 | 100 | 18 | 100 | 104 | CC7 |
| November | 1 | 2.2 | N11 | 219 | 2 | 23 | 15 | 9 | 59 | 1 | 35 | CC4 |
|  |  |  | N110 | 219 | 2 | 23 | 15 | 9 | 59 | 1 | 35 | CC4 |
|  |  |  | N112 | 270 | 108 | 107 | 105 | 138 | 102 | 103 | 105 |  |
|  |  |  | N13 | 219 | 2 | 23 | 15 | 9 | 59 | 1 | 35 | CC4 |
|  |  |  | N14 | 219 | 2 | 23 | 15 | 9 | 59 | 1 | 35 | CC4 |
|  |  |  | N18 | 280 | 117 | 23 | 15 | 9 | 59 | 1 | 35 | CC4 |
|  | 2 | 2.2 | N211 | 206 | 1 | 5 | 14 | 133 | 103 | 107 | 102 |  |
|  |  |  | N23 | 269 | 107 | 36 | 125 | 126 | 47 | 110 | 133 |  |
|  |  |  | N24 | 264 | 103 | 55 | 104 | 102 | 38 | 107 | 107 |  |
|  |  |  | N27 | 232 | 13 | 1 | 123 | 121 | 3 | 1 | 39 |  |
|  |  |  | N28 | 293 | 14 | 49 | 117 | 36 | 57 | 1 | 20 | CC6 |
|  | 5 | 2.8 | N51 | 202 | 1 | 23 | 52 | 47 | 112 | 6 | 130 | CC2 |
|  |  |  | N510 | 214 | 1 | 105 | 112 | 10 | 38 | 1 | 103 |  |
|  |  |  | N513 | 270 | 108 | 107 | 105 | 138 | 102 | 103 | 105 |  |
|  |  |  | N52 | 213 | 1 | 101 | 100 | 47 | 116 | 1 | 144 |  |
|  |  |  | N57 | 241 | 14 | 36 | 6 | 47 | 11 | 1 | 36 |  |
|  | 6 | 2.5 | N612 | 259 | 101 | 41 | 103 | 100 | 18 | 100 | 104 | CC7 |
|  |  |  | N63 | 222 | 3 | 23 | 29 | 87 | 25 | 1 | 12 |  |
|  |  |  | N69 | 254 | 100 | 102 | 106 | 136 | 102 | 106 | 36 |  |
|  | 8 | 16.4 | N82 | 263 | 103 | 5 | 2 | 141 | 103 | 1 | 113 |  |
|  | 10 | 5.7 | N101 | 247 | 14 | 49 | 15 | 47 | 126 | 1 | 108 |  |
| September | 1 | 23.8 | S133 | 223 | 4 | 23 | 28 | 13 | 105 | 1 | 9 |  |
|  |  |  | S139 | 223 | 4 | 23 | 28 | 13 | 105 | 1 | 9 |  |
|  |  |  | S140 | 252 | 14 | 125 | 3 | 111 | 25 | 107 | 135 |  |
|  |  |  | S141 | 201 | 1 | 23 | 52 | 47 | 112 | 6 | 43 | CC2 |
|  |  |  | S143 | 249 | 14 | 48 | 14 | 103 | 109 | 107 | 119 |  |
|  |  |  | S151 | 273 | 110 | 23 | 3 | 105 | 22 | 17 | 66 |  |
|  | 2 | 10.6 | S228 | 238 | 14 | 6 | 29 | 87 | 25 | 1 | 3 | CC5 |
|  |  |  | S229 | 225 | 6 | 6 | 116 | 87 | 18 | 17 | 153 |  |
|  |  |  | S232 | 253 | 20 | 27 | 5 | 3 | 18 | 1 | 4 |  |
|  |  |  | S235 | 265 | 44 | 104 | 28 | 110 | 104 | 1 | 5 |  |
|  |  |  | S238 | 237 | 14 | 1 | 111 | 122 | 47 | 107 | 139 |  |
|  |  |  | S239 | 265 | 44 | 104 | 28 | 110 | 104 | 1 | 5 |  |
|  |  |  | S245 | 278 | 115 | 113 | 14 | 125 | 130 | 107 | 154 |  |
|  |  |  | S247 | 235 | 14 | 1 | 14 | 1 | 19 | 106 | 59 |  |
|  |  |  | S249 | 259 | 101 | 41 | 103 | 100 | 18 | 100 | 104 | CC7 |
|  |  |  | S251 | 255 | 101 | 36 | 102 | 100 | 64 | 100 | 108 |  |
|  |  |  | S254 | 265 | 44 | 104 | 28 | 110 | 104 | 1 | 5 |  |
|  | 3 | 26 | S328 | 223 | 4 | 23 | 28 | 13 | 105 | 1 | 9 |  |
|  |  |  | S332 | 285 | 122 | 113 | 114 | 102 | 47 | 1 | 102 |  |
|  |  |  | S336 | 227 | 6 | 38 | 14 | 144 | 109 | 100 | 100 |  |
|  |  |  | S351 | 229 | 6 | 117 | 14 | 104 | 64 | 1 | 101 |  |
|  | 4 | 7 | S428 | 255 | 101 | 36 | 102 | 100 | 64 | 100 | 108 |  |
|  |  |  | S430 | 283 | 120 | 111 | 14 | 127 | 3 | 9 | 120 |  |
|  |  |  | S438 | 211 | 1 | 100 | 21 | 134 | 64 | 1 | 131 |  |
|  |  |  | S454 | 273 | 110 | 23 | 3 | 105 | 22 | 17 | 66 |  |
|  |  |  | S461 | 262 | 101 | 105 | 14 | 117 | 111 | 117 | 102 |  |
|  | 5 | 14.7 | S529 | 272 | 109 | 120 | 2 | 103 | 124 | 112 | 118 |  |
|  |  |  | S531 | 245 | 14 | 101 | 14 | 69 | 100 | 1 | 103 | CC3 |
|  |  |  | S540 | 236 | 14 | 1 | 46 | 115 | 127 | 105 | 111 |  |
|  |  |  | S541 | 259 | 101 | 41 | 103 | 100 | 18 | 100 | 104 | CC7 |
|  |  |  | S545 | 261 | 101 | 41 | 103 | 100 | 119 | 100 | 104 | CC7 |
|  |  |  | S548 | 265 | 44 | 104 | 28 | 110 | 104 | 1 | 5 |  |
|  |  |  | S553 | 203 | 1 | 42 | 100 | 142 | 115 | 1 | 125 |  |
|  |  |  | S555 | 284 | 53 | 126 | 7 | 143 | 15 | 1 | 136 |  |
|  | 6 | 30.8 | S611 | 209 | 1 | 23 | 4 | 79 | 22 | 17 | 66 |  |
|  |  |  | S620 | 276 | 49 | 109 | 104 | 124 | 38 | 113 | 107 |  |
|  | 8 | 26.5 | S826 | 277 | 114 | 105 | 14 | 104 | 125 | 107 | 101 |  |
|  |  |  | S830 | 201 | 1 | 23 | 52 | 47 | 112 | 6 | 43 | CC2 |
|  | 9 | 35 | S918 | 208 | 1 | 6 | 7 | 87 | 25 | 1 | 132 |  |
|  |  |  | S920 | 242 | 14 | 36 | 46 | 36 | 57 | 1 | 111 | CC6 |
|  |  |  | S926 | 266 | 44 | 115 | 2 | 67 | 2 | 118 | 65 |  |
|  | 10 | 27 | S1024 | 242 | 14 | 36 | 46 | 36 | 57 | 1 | 111 | CC6 |
|  |  |  | S1028 | 205 | 1 | 5 | 3 | 114 | 13 | 1 | 121 |  |
|  |  |  | S1030 | 242 | 14 | 36 | 46 | 36 | 57 | 1 | 111 | CC6 |
|  |  |  | S1036 | 242 | 14 | 36 | 46 | 36 | 57 | 1 | 111 | CC6 |
|  |  |  | S1038 | 228 | 6 | 55 | 7 | 16 | 66 | 8 | 111 |  |
|  |  |  | S1041 | 217 | 1 | 121 | 14 | 129 | 121 | 1 | 39 |  |
|  |  |  | S1042 | 242 | 14 | 36 | 46 | 36 | 57 | 1 | 111 | CC6 |
|  |  |  | S1043 | 201 | 1 | 23 | 52 | 47 | 112 | 6 | 43 | CC2 |
|  | 11 | 34.7 | S1133 | 210 | 1 | 100 | 14 | 130 | 64 | 1 | 142 |  |
|  | 12 | 22.6 | S1223 | 281 | 118 | 122 | 15 | 119 | 65 | 105 | 4 |  |
|  |  |  | S1227 | 216 | 1 | 119 | 21 | 140 | 2 | 6 | 65 |  |

**Supplementary Table S2:** Date of sampling, origin, water salinity during sampling and multi-locus genotype for *Vibrio parahaemolyticus* strains analyzed in this study.

| **Date** | **Site** | **Salinity**  **(**‰**)** | **Isolate** | **Sequence**  **type (ST)** | ***dnaE***  **type** | ***gyrB***  **type** | ***recA***  **type** | ***dtdS***  **type** | ***pntA***  **type** | ***pyrC***  **type** | ***tnaA***  **type** | **Clonal**  **Complex**  **(CC)** |
| --- | --- | --- | --- | --- | --- | --- | --- | --- | --- | --- | --- | --- |
| July | 1 | 28.4 | J1.20 | 2001 | 908 | 915 | 130 | 288 | 18 | 177 | 24 | CC2 |
|  |  |  | J1.25 | 2022 | 237 | 25 | 145 | 905 | 26 | 145 | 116 |  |
|  |  |  | J1.28 | 2008 | 51 | 918 | 75 | 913 | 12 | 37 | 24 | CC5 |
|  |  |  | J1.29 | 2003 | 906 | 16 | 903 | 917 | 26 | 911 | 51 |  |
|  |  |  | J1.41 | 2047 | 12 | 905 | 65 | 903 | 31 | 45 | 66 |  |
|  | 2 | 23 | J2.19 | 2003 | 906 | 16 | 903 | 917 | 26 | 911 | 51 |  |
|  |  |  | J2.26 | 2034 | 71 | 904 | 903 | 908 | 902 | 907 | 902 |  |
|  |  |  | J2.31 | 2010 | 35 | 89 | 98 | 13 | 28 | 5 | 93 |  |
|  |  |  | J2.33 | 481 | 4 | 13 | 11 | 91 | 18 | 9 | 23 |  |
|  |  |  | J2.37 | 2031 | 133 | 901 | 4 | 906 | 43 | 11 | 76 |  |
|  | 3 | 30.3 | J3.3 | 2008 | 51 | 918 | 75 | 913 | 12 | 37 | 24 | CC5 |
|  |  |  | J3.30 | 2013 | 912 | 89 | 914 | 82 | 18 | 904 | 187 |  |
|  |  |  | J3.40 | 2026 | 163 | 909 | 915 | 910 | 903 | 44 | 901 |  |
|  | 4 | 15.4 | J4.16 | 2002 | 907 | 906 | 71 | 35 | 35 | 913 | 23 | CC3 |
|  |  |  | J4.23 | 2000 | 909 | 916 | 910 | 314 | 4 | 280 | 57 |  |
|  |  |  | J4.27 | 2033 | 131 | 221 | 19 | 150 | 114 | 163 | 26 |  |
|  | 5 | 30.2 | J5.13 | 2038 | 51 | 276 | 187 | 5 | 50 | 5 | 167 |  |
|  |  |  | J5.15 | 2009 | 36 | 273 | 921 | 232 | 910 | 27 | 26 | CC6 |
|  |  |  | J5.26 | 2006 | 903 | 914 | 902 | 914 | 50 | 909 | 51 |  |
|  |  |  | J5.29 | 363 | 12 | 180 | 81 | 19 | 21 | 11 | 73 | CC1 |
|  | 6 | 34.4 | J6.26 | 2045 | 19 | 82 | 906 | 150 | 50 | 54 | 33 |  |
|  |  |  | J6.27 | 634 | 7 | 302 | 4 | 26 | 6 | 18 | 26 |  |
|  |  |  | J6.32 | 2052 | 4 | 908 | 911 | 918 | 2 | 109 | 900 |  |
|  |  |  | J6.33 | 481 | 4 | 13 | 11 | 91 | 18 | 9 | 23 |  |
|  | 7 | 36.5 | J7.21 | 634 | 7 | 302 | 4 | 26 | 6 | 18 | 26 |  |
|  |  |  | J7.23 | 2046 | 13 | 349 | 102 | 137 | 26 | 141 | 51 |  |
|  | 8 | 29.3 | J8.15 | 2023 | 196 | 903 | 19 | 29 | 905 | 176 | 61 |  |
|  |  |  | J8.19 | 481 | 4 | 13 | 11 | 91 | 18 | 9 | 23 |  |
|  |  |  | J8.24 | 212 | 69 | 92 | 69 | 114 | 54 | 71 | 24 |  |
|  |  |  | J8.26 | 2039 | 49 | 169 | 67 | 76 | 92 | 906 | 98 |  |
|  |  |  | J8.30 | 481 | 4 | 13 | 11 | 91 | 18 | 9 | 23 |  |
|  |  |  | J8.35 | 2000 | 909 | 916 | 910 | 314 | 4 | 280 | 57 |  |
|  |  |  | J8.38 | 247 | 116 | 149 | 72 | 76 | 45 | 62 | 26 |  |
|  |  |  | J8.39 | 481 | 4 | 13 | 11 | 91 | 18 | 9 | 23 |  |
|  |  |  | J8.41 | 2062 | 4 | 906 | 71 | 35 | 35 | 913 | 23 | CC3 |
|  | 9 | 36.5 | J9.16 | 2014 | 911 | 216 | 97 | 909 | 50 | 263 | 37 |  |
|  | 10 | 35 | J10.14 | 2024 | 178 | 272 | 34 | 206 | 28 | 8 | 24 | CC7 |
|  |  |  | J10.16 | 2025 | 178 | 272 | 918 | 206 | 28 | 8 | 24 | CC7 |
|  |  |  | J10.19 | 2035 | 62 | 82 | 72 | 5 | 1 | 5 | 904 |  |
|  |  |  | J10.20 | 363 | 12 | 180 | 81 | 19 | 21 | 11 | 73 | CC1 |
|  |  |  | J10.25 | 2036 | 61 | 103 | 35 | 110 | 50 | 190 | 73 |  |
|  | 11 | 36.5 | J11.32 | 2032 | 132 | 25 | 908 | 19 | 904 | 901 | 23 |  |
|  | 12 | 23.3 | J12.13 | 2053 | 3 | 136 | 25 | 916 | 909 | 11 | 9 |  |
|  |  |  | J12.16 | 2005 | 904 | 917 | 901 | 38 | 23 | 3 | 12 |  |
|  |  |  | J12.20 | 2007 | 102 | 84 | 34 | 109 | 28 | 107 | 23 | CC4 |
|  |  |  | J12.29 | 131 | 33 | 104 | 96 | 103 | 28 | 11 | 9 |  |
|  |  |  | J12.38 | 2050 | 5 | 136 | 206 | 915 | 912 | 212 | 54 |  |
|  |  |  | J12.44 | 767 | 111 | 239 | 205 | 195 | 28 | 46 | 23 |  |
|  |  |  | J12.45 | 767 | 111 | 239 | 205 | 195 | 28 | 46 | 23 |  |
| November | 2 | 2.2 | N2.10 | 2037 | 60 | 264 | 912 | 217 | 134 | 156 | 141 |  |
|  | 6 | 2.5 | N6.1 | 2027 | 155 | 58 | 65 | 902 | 908 | 11 | 146 |  |
| September | 1 | 23.8 | S1.31 | 2042 | 33 | 918 | 75 | 913 | 12 | 37 | 24 | CC5 |
|  |  |  | S1.42 | 2018 | 902 | 136 | 60 | 180 | 31 | 908 | 54 |  |
|  |  |  | S1.44 | 2019 | 902 | 4 | 911 | 82 | 901 | 903 | 905 |  |
|  |  |  | S1.45 | 2040 | 47 | 133 | 150 | 911 | 26 | 905 | 155 |  |
|  |  |  | S1.48 | 2021 | 900 | 72 | 136 | 89 | 23 | 910 | 23 |  |
|  |  |  | S1.52 | 2005 | 904 | 917 | 901 | 38 | 23 | 3 | 12 |  |
|  | 2 | 10.6 | S2.40 | 2060 | 36 | 273 | 3 | 232 | 910 | 27 | 26 | CC6 |
|  |  |  | S2.41 | 2028 | 154 | 4 | 906 | 920 | 50 | 54 | 23 |  |
|  |  |  | S2.44 | 2006 | 903 | 914 | 902 | 914 | 50 | 909 | 51 |  |
|  |  |  | S2.56 | 467 | 20 | 245 | 31 | 19 | 50 | 157 | 23 |  |
|  | 3 | 26 | S3.29 | 2057 | 3 | 180 | 81 | 19 | 21 | 11 | 73 | CC1 |
|  |  |  | S3.41 | 131 | 33 | 104 | 96 | 103 | 28 | 11 | 9 |  |
|  |  |  | S3.53 | 2002 | 907 | 906 | 71 | 35 | 35 | 913 | 23 | CC3 |
|  | 4 | 7 | S4.33 | 2056 | 3 | 902 | 226 | 82 | 61 | 46 | 86 |  |
|  |  |  | S4.39 | 2004 | 905 | 919 | 25 | 13 | 911 | 3 | 94 |  |
|  |  |  | S4.49 | 2004 | 905 | 919 | 25 | 13 | 911 | 3 | 94 |  |
|  |  |  | S4.53 | 2044 | 19 | 907 | 916 | 232 | 142 | 902 | 24 |  |
|  | 5 | 14.7 | S5.33 | 2010 | 35 | 89 | 98 | 13 | 28 | 5 | 93 |  |
|  |  |  | S5.35 | 2029 | 152 | 106 | 17 | 13 | 99 | 171 | 26 |  |
|  |  |  | S5.61 | 2020 | 901 | 72 | 34 | 89 | 61 | 910 | 24 |  |
|  | 6 | 30.8 | S6.17 | 2005 | 904 | 917 | 901 | 38 | 23 | 3 | 12 |  |
|  |  |  | S6.23 | 2041 | 42 | 911 | 200 | 75 | 912 | 46 | 24 |  |
|  | 7 | 34.6 | S7.14 | 2030 | 137 | 235 | 909 | 918 | 18 | 241 | 86 |  |
|  | 8 | 26.5 | S8.29 | 2000 | 909 | 916 | 910 | 314 | 4 | 280 | 57 |  |
|  | 9 | 35 | S9.25 | 2012 | 913 | 920 | 900 | 120 | 23 | 238 | 26 |  |
|  |  |  | S9.27 | 2015 | 910 | 921 | 31 | 50 | 907 | 116 | 127 |  |
|  | 10 | 27 | S10.26 | 2063 | 102 | 84 | 31 | 109 | 28 | 107 | 23 | CC4 |
|  |  |  | S10.35 | 2000 | 909 | 916 | 910 | 314 | 4 | 280 | 57 |  |
|  |  |  | S10.37 | 2050 | 5 | 136 | 206 | 915 | 912 | 212 | 54 |  |
|  |  |  | S10.44 | 2053 | 3 | 136 | 25 | 916 | 909 | 11 | 9 |  |
|  |  |  | S10.47 | 2011 | 913 | 912 | 209 | 904 | 28 | 32 | 192 |  |
|  | 11 | 34.7 | S11.26 | 537 | 119 | 151 | 197 | 79 | 4 | 205 | 105 |  |
|  |  |  | S11.27 | 2017 | 909 | 117 | 907 | 907 | 906 | 900 | 132 |  |
|  |  |  | S11.36 | 2043 | 31 | 345 | 246 | 901 | 18 | 11 | 57 |  |
|  |  |  | S11.38 | 114 | 55 | 15 | 31 | 55 | 18 | 58 | 46 |  |
|  | 12 | 22.6 | S12.25 | 2049 | 6 | 252 | 39 | 267 | 155 | 243 | 24 |  |
|  |  |  | S12.31 | 411 | 2 | 113 | 72 | 94 | 26 | 83 | 23 |  |
|  |  |  | S12.41 | 2058 | 908 | 915 | 19 | 288 | 18 | 177 | 24 | CC2 |

**Supplementary Table S3:** Genetic parameters of *Vibrio cholerae* isolates (n=109), in July (n=36), in September (n=52), in November (n=21) and in conditions of high (n=61) or low salinity (n=48).

| **Locus (size in bp)** | **group** | **Number of alleles / strain** | **Genetic diversity**  **(h or H**^a^**)** | **Number of polymorphic sites (%)** | **Number of non-synomynous codon** | **dN**^b^ | **dS**^c^ | **dN/dS** |
| --- | --- | --- | --- | --- | --- | --- | --- | --- |
| ***adk* (416)** | Total | 0.25 | 0.8862 | 27 (6.49%) | 4 | 0.0037 | 0.0267 | 0.1386 |
|  | July | 0.31 | 0.8571 | 10 (2.40%) | 2 | 0.0033 | 0.0194 | 0.1701 |
|  | September | 0.31 | 0.8824 | 19 (4.57%) | 2 | 0.0035 | 0.0287 | 0.1220 |
|  | November | 0.52 | 0.9190 | 13 (3.13%) | 2 | 0.0052 | 0.0295 | 0.1763 |
|  | High salinity | 0.30 | 0.8604 | 16 (3.85%) | 2 | 0.0034 | 0.0209 | 0.1627 |
|  | Low salinity | 0.40 | 0.9211 | 23 (5.53%) | 3 | 0.0039 | 0.0347 | 0.1124 |
| ***gyrB* (431)** | Total | 0.32 | 0.9344 | 40 (9.28%) | 1 | 0.0031 | 0.0556 | 0.0558 |
|  | July | 0.44 | 0.9016 | 17 (3.94%) | 1 | 0.0030 | 0.0290 | 0.1034 |
|  | September | 0.50 | 0.9434 | 29 (6.72%) | 1 | 0.003 | 0.0335 | 0.0896 |
|  | November | 0.52 | 0.8810 | 25 (5.80%) | 1 | 0.010 | 0.0603 | 0.1658 |
|  | High salinity | 0.43 | 0.9305 | 24 (5.57%) | 1 | 0.0030 | 0.0303 | 0.0990 |
|  | Low salinity | 0.44 | 0.9415 | 35 (8.12%) | 1 | 0.0033 | 0.0758 | 0.0435 |
| ***mdh* (421)** | Total | 0.28 | 0.9269 | 33 (7.84%) | 0 | - | - | - |
|  | July | 0.42 | 0.8841 | 25 (5.94%) | 0 | - | - | - |
|  | September | 0.37 | 0.9178 | 18 (4.28%) | 0 | - | - | - |
|  | November | 0.71 | 0.9238 | 21 (4.99%) | 0 | - | - | - |
|  | High salinity | 0.33 | 0.9234 | 26 (6.18%) | 0 | - | - | - |
|  | Low salinity | 0.50 | 0.9504 | 25 (5.94%) | 0 | - | - | - |
| ***metE* (591)** | Total | 0.48 | 0.9659 | 109 (18.44%) | 16 | 0.0041 | 0.1675 | 0.0245 |
|  | July | 0.56 | 0.9540 | 73 (12.35%) | 7 | 0.0039 | 0.1646 | 0.0237 |
|  | September | 0.62 | 0.9713 | 79 (13.36%) | 9 | 0.0038 | 0.1539 | 0.0247 |
|  | November | 0.62 | 0.9190 | 87 (14.72%) | 9 | 0.0054 | 0.2285 | 0.0236 |
|  | High salinity | 0.56 | 0.9662 | 81 (13.71%) | 10 | 0.0037 | 0.1574 | 0.0235 |
|  | Low salinity | 0.38 | 0.9610 | 100 (16.92%) | 12 | 0.0047 | 0.1804 | 0.0261 |
| ***pntA* (431)** | Total | 0.39 | 0.9709 | 38 (8.82%) | 4 | 0.0034 | 0.0425 | 0.0800 |
|  | July | 0.58 | 0.9556 | 26 (6.03%) | 2 | 0.0032 | 0.0454 | 0.0705 |
|  | September | 0.52 | 0.9646 | 26 (6.03%) | 3 | 0.0033 | 0.0398 | 0.0829 |
|  | November | 0.62 | 0.9286 | 26 (6.03%) | 2 | 0.0046 | 0.043 | 0.1070 |
|  | High salinity | 0.46 | 0.9675 | 29 (6.73%) | 3 | 0.0033 | 0.0408 | 0.0809 |
|  | Low salinity | 0.54 | 0.9645 | 33 (7.66%) | 2 | 0.0037 | 0.0453 | 0.0817 |
| ***purM* (476)** | Total | 0.17 | 0.6984 | 62 (13.03%) | 0 | - | - | - |
|  | July | 0.22 | 0.6397 | 15 (3.15%) | 0 | - | - | - |
|  | September | 0.25 | 0.7632 | 19 (3.99%) | 0 | - | - | - |
|  | November | 0.33 | 0.6190 | 55 (11.55%) | 0 | - | - | - |
|  | High salinity | 0.20 | 0.7136 | 19 (3.99%) | 0 | - | - | - |
|  | Low salinity | 0.25 | 0.7119 | 59 (12.39%) | 0 | - | - | - |
| ***pyrC* (449)** | Total | 0.46 | 0.9743 | 105 (23.39%) | 18 | 0.0108 | 0.1854 | 0.0583 |
|  | July | 0.61 | 0.9571 | 85 (18.93%) | 16 | 0.0121 | 0.2252 | 0.0537 |
|  | September | 0.58 | 0.9668 | 75 (16.70%) | 15 | 0.0105 | 0.1595 | 0.0658 |
|  | November | 0.71 | 0.9429 | 93 (20.71%) | 15 | 0.0105 | 0.1877 | 0.0559 |
|  | High salinity | 0.52 | 0.9708 | 87 (19.38%) | 17 | 0.0105 | 0.199 | 0.0528 |
|  | Low salinity | 0.63 | 0.9725 | 94 (20.94%) | 15 | 0.0114 | 0.1724 | 0.0661 |
| **All loci**  **(3215)** | Total | 0.72 | 0.9082 | 414 (13.25%) | 43 | 0.9082 | 0.0022 | 0.074 |
|  | July | 0.75 | 0.8785 | 251 (8.03%) | 28 | 0.8785 | 0.0021 | 0.0717 |
|  | September | 0.73 | 0.9156 | 265 (8.48%) | 30 | 0.9156 | 0.0023 | 0.0669 |
|  | November | 0.81 | 0.8762 | 320 (10.24%) | 29 | 0.8762 | 0.0024 | 0.0983 |
|  | High salinity | 0.72 | 0.8942 | 282 (9.02%) | 33 | 0.8942 | 0.0021 | 0.0691 |
|  | Low salinity | 0.79 | 0.9176 | 369 (11.81%) | 33 | 0.9176 | 0.0024 | 0.0812 |

^a^H is for mean genetic diversity calculated for all loci. ^b^dN = non-synonymous substitutions per non-synonymous site. *^c^*dS = synonymous substitutions per synonymous site.

**Supplementary Table S4:** Genetic parameters of *Vibrio parahaemolyticus* isolates (n=89), in July (n=49), in September (n=38) and in conditions of high (n=73) or low salinity (n=16).

| **Locus (size in bp)** | **group** | **Number of alleles / strain** | **Genetic diversity (h or H**^a^**)** | **Number of polymorphic site (%)** | **Number of non-synomynous codon** | **dN**^b^ | **dS**^c^ | **dN/dS** |
| --- | --- | --- | --- | --- | --- | --- | --- | --- |
| ***dnaE* (557)** | Total | 0.60 | 0.9837 | 127 (22.80%) | 16 | 0.0045 | 0.1173 | 0.0384 |
|  | July | 0.69 | 0.9728 | 38 (6.82%) | 1 | 0.0023 | 0.0574 | 0.0401 |
|  | September | 0.76 | 0.9917 | 115 (20.65%) | 16 | 0.0023 | 0.0574 | 0.0401 |
|  | High salinity | 0.62 | 0.9806 | 54 (9.69%) | 7 | 0.003 | 0.0586 | 0.0512 |
|  | Low salinity | 0.94 | 0.9844 | 106 (19.03%) | 11 | 0.0121 | 0.4057 | 0.0298 |
| ***gyrB* (592)** | Total | 0.61 | 0.9860 | 63 (10.64%) | 4 | 0.0088 | 0.1045 | 0.0842 |
|  | July | 0.67 | 0.9813 | 46 (7.77%) | 0 | - | - | - |
|  | September | 0.82 | 0.9917 | 44 (7.43%) | 4 | 0.0088 | 0.1032 | 0.0853 |
|  | High salinity | 0.63 | 0.9836 | 60 (10.14 %) | 4 | 0.0088 | 0.1047 | 0.0840 |
|  | Low salinity | 0.94 | 0.9886 | 29 (4.90%) | 0 | - | - | - |
| ***tnaA* (423)** | Total | 0.38 | 0.9852 | 27 (6.38%) | 1 | 0.0030 | 0.0430 | 0.0698 |
|  | July | 0.47 | 0.9852 | 21 (4.96%) | 0 | - | - | - |
|  | September | 0.50 | 0.9917 | 16 (3.78%) | 1 | 0.003 | 0.0453 | 0.0662 |
|  | High salinity | 0.42 | 0.9804 | 26 (6.15%) | 1 | 0.003 | 0.043 | 0.0698 |
|  | Low salinity | 0.63 | 0.9886 | 11 (2.60%) | 0 | - | - | - |
| ***recA* (729)** | Total | 0.56 | 0.9847 | 150 (20.71%) | 16 | 0.0034 | 0.1619 | 0.0210 |
|  | July | 0.67 | 0.9796 | 65 (8.91%) | 10 | 0.0030 | 0.1128 | 0.0266 |
|  | September | 0.82 | 0.9844 | 139 (19.07%) | 7 | 0.0039 | 0.2195 | 0.0178 |
|  | High salinity | 0.62 | 0.9852 | 150 (20.58%) | 16 | 0.0038 | 0.1750 | 0.0217 |
|  | Low salinity | 0.94 | 0.9829 | 45 (6.17%) | 0 | 0.002 | 0.111 | 0.0180 |
| ***pyrC* (493)** | Total | 0.55 | 0.9512 | 41 (8.32%) | 7 | 0.0036 | 0.0511 | 0.0705 |
|  | July | 0.65 | 0.9414 | 32 (6.49%) | 5 | 0.004 | 0.0494 | 0.0810 |
|  | September | 0.76 | 0.9583 | 33 (6.69%) | 5 | 0.0031 | 0.0532 | 0.0583 |
|  | High salinity | 0.60 | 0.9464 | 39 (7.91%) | 6 | 0.0037 | 0.0517 | 0.0716 |
|  | Low salinity | 0.94 | 0.9602 | 22 (4.46%) | 4 | 0.0032 | 0.0479 | 0.0668 |
| ***pntA* (430)** | Total | 0.39 | 0.9747 | 29 (6.74%) | 4 | 0.0031 | 0.0389 | 0.0797 |
|  | July | 0.51 | 0.9741 | 24 (5.58%) | 3 | 0.0031 | 0.0365 | 0.0849 |
|  | September | 0.55 | 0.9917 | 21 (4.88%) | 2 | 0.0031 | 0.043 | 0.0721 |
|  | High salinity | 0.38 | 0.9745 | 27 (6.28%) | 4 | 0.0031 | 0.0381 | 0.0814 |
|  | Low salinity | 0.75 | 0.9787 | 15 (3.49%) | 1 | 0.003 | 0.0434 | 0.0691 |
| ***dtdS* (458)** | Total | 0.55 | 0.9308 | 62 (13.54%) | 3 | 0.0101 | 0.4641 | 0.0218 |
|  | July | 0.67 | 0.933 | 57 (12.45%) | 3 | 0.0101 | 0.4603 | 0.0219 |
|  | September | 0.76 | 0.9333 | 37 (8.08%) | 0 | - | - | - |
|  | High salinity | 0.63 | 0.9226 | 60 (13.10%) | 3 | 0.0101 | 0.4647 | 0.0217 |
|  | Low salinity | 0.75 | 0.9417 | 31 (6.77%) | 0 | - | - | - |
| **All loci**  **(3682)** | Total | 0.76 | 0.9705 | 499 (13.55%) | 51 | 0.9705 | 0.0012 | 0.0717 |
|  | July | 0.80 | 0.9654 | 283 (7.69%) | 22 | 0.9654 | 0.001 | 0.0673 |
|  | September | 0.92 | 0.9744 | 405 (11.00%) | 35 | 0.9744 | 0.0015 | 0.0775 |
|  | High salinity | 0.78 | 0.9689 | 416 (11.30%) | 41 | 0.9689 | 0.0011 | 0.07 |
|  | Low salinity | 0.94 | 0.9714 | 259 (7.03%) | 16 | 0.9714 | 0.0016 | 0.0809 |

^a^H is for mean genetic diversity calculated for all loci. ^b^dN = non-synonymous substitutions per non-synonymous site. *^c^*dS = synonymous substitutions per synonymous site.

**Supplementary Table S5:** Main results from non-parametric (Wilcoxon) and ANOVA analyses of links between genetic data of *V. cholerae* and *V. parahaemolyticus* strains and, date of sampling (July, September, November 2011), sampling stations (lagoon, beach, channel between river and sea, and channel between lagoon and sea) and water salinity. Grey cells showed incongruous results between Wicoxon and ANOVA analyses.

**Supplementary Table S6:** Gene and percentage of strain implicated in recombination by group for *V. cholerae* population. Reliability of recombination events detection represents number of methods detecting significatively the recombination event. RS/n corresponds to the percentage of strain implicated in recombination related to strain in the group.

| Reliability of recombination events detection | Total  (n=109) | | July  (n=36) | | September  (n=52) | | November  (n=21) | | High salinity  (n=61) | | Low salinity  (n=48) | |
| --- | --- | --- | --- | --- | --- | --- | --- | --- | --- | --- | --- | --- |
|  | RS/n (%) | gene | RS/n (%) | gene | RS/n (%) | gene | RS/n (%) | gene | RS/n (%) | gene | RS/n (%) | gene |
| 7 | 8.3% | *pyrC* | 11.1% | *pyrC* | 7.7% | *pyrC* | 23.8% | *pyrC* | 8.2% | *pyrC* | 8.3% | *pyrC* |
| 6 | 16.5% | *pyrC metE* | 13.9% | *pyrC metE* | 19.2% | *metE* | 14.3% | *metE* | 18.0% | *pyrC metE* | 14.6% | *metE* |
| 5 | 1.8% | *metE* | 2.8% | *metE* | 1.9% | *metE* | 0% | *-* | 1.6% | *metE* | 2.1% | *metE* |
| 4 | 9.2% | *pyrC metE pntA* | 13.9% | *pyrC metE pntA* | 7.7% | *pyrC metE pntA* | 4.8% | *pyrC* | 13.1% | *pyrC metE pntA* | 4.2% | *pyrC metE pntA* |
| total | 31.2% | *pyrC metE pntA* | 41.7% | *pyrC metE pntA* | 36.5% | *pyrC metE pntA* | 42.9% | *pyrC metE* | 41.0% | *pyrC metE pntA* | 25.0% | *pyrC metE pntA* |

**Supplementary Table S7:** Gene and percentage of strain implicated in recombination by group for *V. parahaemolyticus* population. Reliability of recombination events detection represents number of methods detecting significatively the recombination event. RS/n corresponds to the percentage of strain implicated in recombination related to strain in group.

| Reliability of recombination events detection | Total  (n=89) | | July  (n=49) | | September  (n=38) | | November  (n=2) | | High salinity  (n=73) | | Low salinity  (n=16) | |
| --- | --- | --- | --- | --- | --- | --- | --- | --- | --- | --- | --- | --- |
|  | RS/n (%) | gene | RS/n (%) | gene | RS/n (%) | gene | RS/n (%) | gene | RS/n (%) | gene | RS/n (%) | gene |
| 7 | 15.7% | *recA dtdS* | 18.4% | *recA dtdS* | 10.5% | *recA* | 50% | *recA* | 15.1% | *recA dtdS* | 18.8% | *recA* |
| 6 | 40.4% | *recA* | 32.7% | *recA* | 50.0% | *recA* | 50% | *recA* | 42.5% | *recA* | 31.3% | *recA* |
| 5 | 2.2% | *dnaE recA* | 2.0% | *recA* | 26% | *dnaE* | 0% | *-* | 1.4% | *recA* | 6.3% | *dnaE* |
| 4 | 0% | *-* | 0% | *-* | 0% | *-* | 0% | *-* | 0% | *-* | 0% | *-* |
| total | 58.4% | *recA dtdS dnaE* | 53.1% | *recA dtdS* | 63.2% | *recA dnaE* | 100% | *recA* | 58.9% | *recA dtdS* | 56.3% | *recA dnaE* |


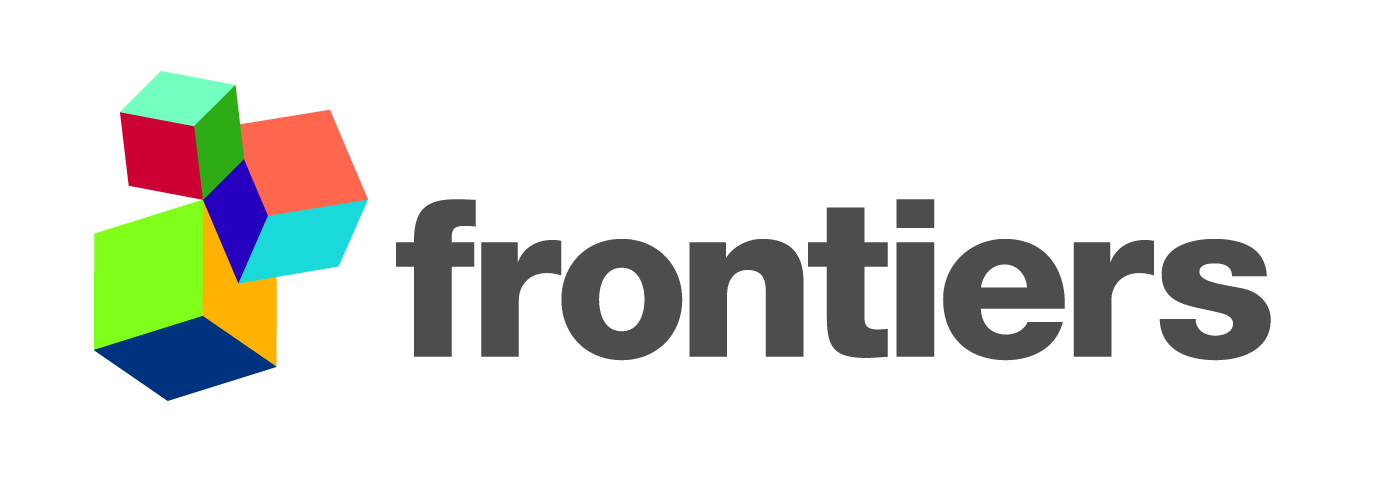

Supplement: Supplementary file 1 [file DataSheet1.DOCX]
